# Single sarcomere contraction dynamics in a whole muscle

**DOI:** 10.1038/s41598-018-33658-7

**Published:** 2018-10-15

**Authors:** Eng Kuan Moo, Walter Herzog

**Affiliations:** Human Performance Laboratory, Faculty of Kinesiology, University of Calgary, Calgary, Alberta Canada

## Abstract

The instantaneous sarcomere length (SL) is regarded as an important indicator of the functional properties of striated muscle. Previously, we found greater sarcomere elongations at the distal end compared to the mid-portion in the mouse tibialis anterior (TA) when the muscle was stretched passively. Here, we wanted to see if SL dispersions increase with activation, as has been observed in single myofibrils, and if SL dispersions differ for different locations in a muscle. Sarcomere lengths were measured at a mid- and a distal location of the TA in live mice using second harmonic generation imaging. Muscle force was measured using a tendon force transducer. We found that SL dispersions increased substantially from the passive to the active state, and were the same for the mid- and distal portions of TA. Sarcomere length non-uniformities within a segment of ~30 serial sarcomeres were up to 1.0 µm. We conclude from these findings that passive, mean SLs obtained from a single location are not necessarily representative of the distribution of SL in active muscle, and thus may be misinterpreted when deriving muscle mechanical properties, such as the force-length relationship. In view of these findings, it seems crucial to determine how SL distributions within a muscle relate to the most fundamental properties of muscle, such as the maximal isometric force.

## Introduction

Skeletal muscles produce forces through tens of thousands of sarcomeres that are hierarchically organized within a complex network of connective tissues. Sarcomeres are considered as the most basic contractile units in muscles. They are made up of the contractile myofilaments (myosin and actin) and a series of structural proteins (e.g., titin, nebulin and desmin). The thick (myosin) and thin (actin) myofilaments give skeletal muscles the striated appearance that can be observed with light microscopy. Based on the sliding filament^1,2^ and cross-bridge^3,4^ theories, a sarcomere produces force through the actin-myosin interaction. The maximal isometric force that can be generated by sarcomeres depends on the amount of overlap between the thick and thin myofilaments, which can be predicted using the theoretical force-length (FL) relationship^3,5^. The instantaneous sarcomere length (SL) and the rate of change in SL are regarded as important indicators of the functional properties (e.g., force, power) of a muscle^3,6–8^.

Sarcomere lengths (SLs) can be measured easily in single myofibers and myofibrils using phase-contrast light microscopy. The contractile behaviour of sarcomeres has been studied extensively using single myofibers^3,9,10^ and myofibrils^11–14^ in an attempt to unravel fundamental properties of muscle contraction and function. An important observation in isolated myofibers and myofibrils is the non-uniform sarcomere lengths in active *in vitro* preparations^7,15,16^. These sarcomere length non-uniformities have been thought to be the cause of a variety of muscle properties and functions, for example, the residual force enhancement and force depression properties^17^, and the loss of force following eccentric contractions^18,19^. However, there is an intricate structural connective tissue network in the whole muscle that can provide stability to the sarcomeres^20,21^. It is unknown if the behavior of sarcomere length non-uniformities observed in fibers and myofibrils is representative of sarcomeres in whole muscle or it is just an artifact in isolated fibers and myofibrils that is not present in entire muscles.

With recent advances of non-linear laser microscopy, individual sarcomeres can now be visualized and their lengths measured accurately in intact whole muscles^21–25^. Using such techniques, it has been found that SL dispersions (also referred to as SL non-uniformities) in intact relaxed muscles^21^ are comparable to those found in single muscle fibers/fibrils^11,13,26^. However, little is known regarding SL in actively contracting whole muscles. The active properties of sarcomeres are arguably more important than the corresponding passive properties, particularly when determining mechanical properties of muscles, such as the FL relationship. In a recent study, we found that the SLs measured in an active muscle gave better predictions of the maximal isometric force than the corresponding SLs measured in the passive muscle^25^. However, SLs were measured only in the mid-belly of the muscle and there is evidence suggesting that SLs may depend on the location of sarcomeres within a muscle^21^. Thus, it remains to be explored what SL non-uniformities may exist at different locations within a given muscle, and how they may differ between a passive and an active muscle.

The purpose of this study was to measure and compare the contractile behaviours of sarcomeres at different locations in an intact whole muscle. Sarcomere lengths were measured at a mid- and a distal location of the tibialis anterior (TA) in live mice. We used multi-photon excitation microscopy to visualize sarcomeres in whole muscle, and a modified buckle-type tendon force transducer^27^ to directly measure the forces generated in the mouse tibialis anterior muscle. We hypothesized that sarcomere contractile behaviour depends on the location of the sarcomeres within the TA muscle.

## Methods

### Animal preparation

All aspect of animal care and experimental procedures were carried out in accordance with the guidelines of the Canadian Council on Animal Care and were approved by the University of Calgary Life Sciences Animal Research and Ethics Committee. Sarcomere lengths were measured at either the mid- or distal location of the TA. A total of seventeen 10–12 week-old male C57BL6 mice were used and randomly assigned into the mid-TA (n = 9) and distal TA (n = 8) groups. Animals were anesthetized using a 1–2% isoflurane/oxygen mixture. The core temperature of the mice was maintained at ~30 °C. The fascial plane between the gluteus maximus and the anterior head of the biceps femoris was opened for implantation of a cuff-type bipolar electrode^28^ of ~0.8 mm-diameter on the sciatic nerve. The left proximal femur was fixed using a custom-made clamp at a knee flexion angle of approximately 120° (full knee extension = 180°), while the left foot was pinned to a movable base to allow for changing the ankle joint angle, thereby adjusting the TA muscle tendon unit (MTU) length. The skin over the left TA was opened and stretched to form a pool to accommodate a phosphate buffered saline solution that kept the muscle hydrated and allowed for imaging using a water-immersion objective. The fascia over the TA was removed. All distal tendons around the ankle, except for the tendon of the TA, were severed to eliminate the influence of other muscle groups during force measurement and to allow for a full range of motion of the ankle. A custom-made E-shaped tendon force transducer^29^ was implanted onto the distal TA tendon to measure TA force.

### Imaging of *in vivo* sarcomeres in relaxed and activated muscle at isometric lengths

**Figure 1:**
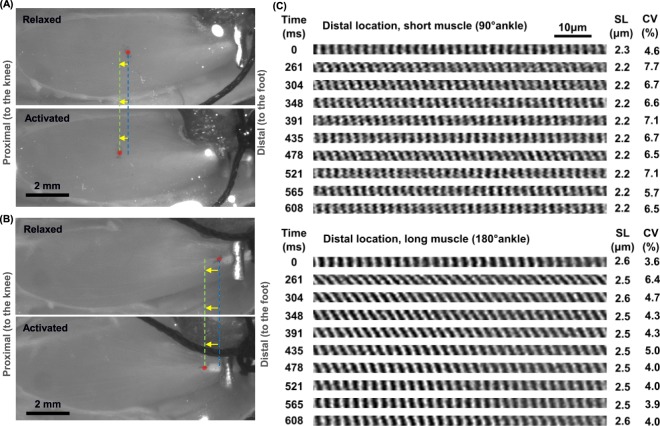
Digital photographs of mice tibialis anterior (TA) muscle prepared for second harmonic generation (SHG) imaging of *in vivo* sarcomeres at the mid- (**A**) and distal (**B**) TA. When activated, the mid- and distal TA (marked by the red circle) were displaced proximally by ~375 µm (**A**) and ~600 µm (**B**), respectively. Two fluorescent markers (not visible in the images) that were separated by 1 mm were applied at the mid- or distal TA (in proximity to the red circle) and observed under fluorescent light to measure the local displacement of the muscle caused by activation. (**C**) Representative time-series of the two-dimensional image bands of sarcomeres in the relaxed (0 ms) and the activated states at the distal TA at the short (top) and the long (bottom) lengths. The A-bands appear as white bands in the images. The mean sarcomere length (SL) and coefficients of variation (CV) of SLs are shown for each image band. Note that the image bands acquired at time 215 ms were excluded due to the image quality not meeting the selection criteria described in the Methods’ section.

The imaging protocol used in this study has been described in detail previously^25^. Briefly, two fluorescent markers separated by ~1 mm were attached to the mid- or distal region of the muscle using a 100µm-diameter glass tip attached to a 3-axes linear micro-manipulator (Newport Corp., CA, USA). A scanning area was selected between the two fluorescent markers in the relaxed muscle. The TA was held at isometric lengths and was activated using a 600 ms, continuous, supra-maximal electrical stimulation (3 × α-motor neuron threshold, 60–70 Hz, 0.1 ms square wave pulse) of the sciatic nerve. A stimulation frequency that elicits near-maximal force, but yet induces minimal muscle fatigue, was chosen. This stimulation frequency varies among animals but typically ranges between 60–70 Hz. During activation, muscle fibers moved proximally (Fig. 1). This activation-induced displacement was measured using the two fluorescent markers and a multi-photon excitation microscope (FVMPE-RS model, Olympus, Tokyo, Japan) (see Supplementary Material, S1 for details of the displacement measurements and correction). Sarcomeres were imaged in the target area of the TA muscle in its relaxed and active states.

Sarcomeres were visualized by second harmonic generation (SHG) imaging of the TA using an upright, multi-photon excitation microscope (FVMPE-RS model, Olympus, Tokyo, Japan) equipped with a wavelength-tunable (680–1300 nm), ultrashort-pulsed (pulse width: <120 fs; repetition rate: 80 MHz) laser (InSight DeepSee-OL, Spectra-Physics, CA, USA) and a 25×/1.05 NA water immersion objective (XLPLN25XWMP2 model, Olympus, Tokyo, Japan). The laser wavelength was 800 nm. The resulting SHG signal emitted by the muscle was collected in the backward (epi-) direction using a band-pass filter at the harmonic frequency (FF01 400/40, Semrock Inc. NY, USA). The average laser power in the sample plane was adjusted between 15–18 mW to ensure optimal imaging without thermal damage to the muscle.

Time-series images were acquired in the horizontal plane (imaging area: 159 µm × 2.8 µm; pixel size: 0.2 µm; bit-depth: 12; dwell time: 2 µs) at a frame rate of 23 frames/s. Images were taken from the top 100 µm of the TA, as SLs have been shown to be similar across the depth of the muscle^21,30^. The SHG imaging of the muscle was performed at ankle angles of 90° and 180° (full plantarflexion) which corresponded to, and will hereafter be denoted as, short and long MTU length, respectively.

### Image analysis and sarcomere length measurement

The imaging/stimulation trials were divided into four groups based on the muscle states (relaxed vs. activated) and the MTU lengths (short vs. long): (1) relaxed-short (49 trials in mid-TA group; 46 trials in distal TA group); (2) activated-short (120 trials in mid-TA group; 169 trials in distal TA group); (3) relaxed-long (47 trials in mid-TA group; 44 trials in distal TA group); (4) activated-long (174 trials in mid-TA group; 159 trials in distal TA group).

Only images with good signal-to-noise ratio and minimal motion artifact were included for image analysis. For the activated muscle, only images acquired during the steady-state phase of the near- maximal contraction, which includes images acquired between 200–600 ms of the contraction, were analyzed. All images that fit the aforementioned inclusion criteria were processed by the image processing tools.

Planar image bands of 2.8µm-width that contained 5–30 sarcomeres in series were selected. Considering that the diameter of a myofibril is ~1.3 µm^31^, each analyzed image band was estimated to contain approximately two parallel-running myofibrils, and therefore contain between 10–60 sarcomeres. The selected image bands were band-pass-filtered using Fiji software (National Institutes of Health, MD, USA), and were processed using a custom-written MATLAB code that identified the centroids of the sarcomeric A-bands. Individual SLs were measured as the distance between adjacent A-band centroids^21,23^. As mouse TA has a spindle-like shape, the surface tilt of the local muscle region was measured using a through-thickness muscle image, and SLs were corrected for out-of-plane orientation (see Supplementary Material, S2, for details).

At the end of the image processing, the variables of interest including the mean, standard deviation (SD), coefficient of variation (CV = standard deviation/mean), shortest, longest, and length range (the difference between the longest and the shortest sarcomere in an image band) of SLs were derived from each image band. We grouped the data by contraction time and quantified possible changes of the mean values of the mean, CV, and range of SLs, as a function of contraction time.

In every imaging/stimulation trial in the activated muscle, ten time-series images that were acquired between 200–600 ms following the onset of contraction were processed. The SLs measured from these time-series images were similar (see Results), and therefore, were pooled for subsequent analysis. In total, the number of image bands analyzed in the relaxed-short, activated-short, relaxed-long, activated-long groups were 144, 505, 132, 1002 bands, respectively, for the mid-TA group, and 257, 1021, 235, 983 bands, respectively, for the distal TA group. We report the average values of the mean, SD, CV, shortest, longest, and length range of SLs measured from all image bands acquired from the nine and eight animals in the mid-TA group and distal TA group, respectively.

From the pooled SLs in the mid-TA and the distal TA groups, a probability distribution function (PDF) was determined for each of the relaxed-short, activated-short, relaxed-long and activated-long categories. Two weighted Gaussian curves were fitted to individual PDFs using a Gaussian mixture model with variation inference^32^.

Finally, we categorized the individual SLs as belonging to the ascending limb, plateau, and descending limb of the theoretical sarcomere FL curve, in order to study the activation-induced changes in SL distribution on the FL curve.

### Statistical analysis

Statistical analyses were performed using SPSS (version 22, SPSS Inc. IL, USA). Unless otherwise stated, the results were expressed as estimated marginal means (EMM) ± standard error. The means of the mean, local SD, local CV, longest, shortest, and length range of SLs were analyzed for condition effects, which include muscle states (relaxed vs stimulated), muscle lengths (short vs long) and location on the muscles (mid-TA vs distal TA), using a generalized estimating equation (GEE, under Genlin procedures in SPSS) to take into account the correlated nature of the observations and the unbalanced study design. The relationships between contraction time and the mean SL, local CV, and length range of SLs were also individually analyzed with GEE. All statistical tests are two-sided with type I error, α, set at 0.05 level. Multiple comparisons were accounted for through Bonferroni adjusted *p*-values. As long as the data were interval-type, no assumptions regarding normal distribution was needed for the GEE statistical method.

## Results

The average mass of the mice in the mid-TA and distal TA groups was 29 ± 4 g and 28 ± 3 g (mean ± SD), respectively. The total number of sarcomeres analyzed for each of the ‘relaxed-short’, ‘activated-short’, ‘relaxed-long’, and ‘activated-long’ categories was, respectively, 2,856 sarcomeres, 4,454 sarcomeres, 2,564 sarcomeres, and 8,270 sarcomeres in the mid-TA group, and was, respectively, 4,611 sarcomeres, 7,778 sarcomeres, 3,537 sarcomeres, and 7,458 sarcomeres in the distal TA group. Muscle activation led to a proximal displacement of the mid- and distal TA target sites of 338 ± 56 µm and 587 ± 32 µm (mean ± SD), respectively.

**Figure 2:**
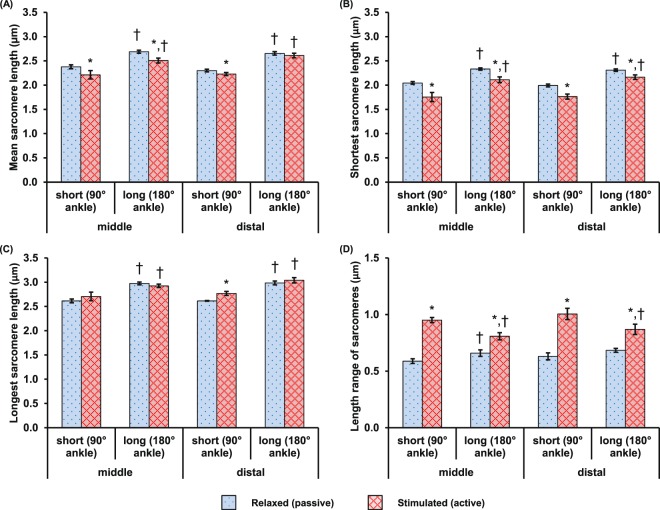
Mean values of (**A**) the mean sarcomere length (SL), (**B**) the shortest SL, (**C**) the longest SL and, (**D**) the SL range, which is defined as the difference between the longest and the shortest sarcomere in individual image bands, measured at the mid- and distal TA. The data were pooled from 9 animals for the mid-TA group and 8 animals for the distal TA group, respectively. During activation, sarcomeres at the mid-TA shortened by 6.7–7.1%, while sarcomeres at the distal TA shortened by 0–3% (**A**). The shortest SL in individual image bands decreased (**B**), but the longest SL in individual image bands remained unchanged (**C**) during activation. The overall effect was an increase of the SL range during activation (**D**). Note that there is no statistical difference between the results at the mid-TA and distal TA regions. *Indicates significant differences in mean SL, shortest SL, longest SL or SL range compared with sarcomeres in the relaxed muscle at either the short or long length (*p* < 0.01). ^†^Indicates significant differences in mean SL, shortest SL, longest SL or SL range compared with sarcomeres in either the relaxed or the activated muscle at the short length (*p* < 0.01).

The mean, shortest, longest, and length range of SLs measured at the mid- and distal TAs showed no statistical difference (Fig. 2). Sarcomeres in the relaxed-short (2.4 µm in the mid-TA; 2.3 µm in the distal TA) muscle were shorter than the sarcomeres in the relaxed-long (2.7 µm in both the mid- and distal TA) muscle. During activation, sarcomeres in the mid-TA shortened by 6.7–7.1% and sarcomeres in the distal TA shortened by 0–3% (Fig. 2A). Activation led to a decrease in the shortest SL but no changes in the longest SL in the individual image bands (Fig. 2B,C). Therefore, the SL range in individual image bands increased from ~0.6 µm in the relaxed to ~0.9 µm in the activated muscles (Fig. 2D).

**Figure 3:**
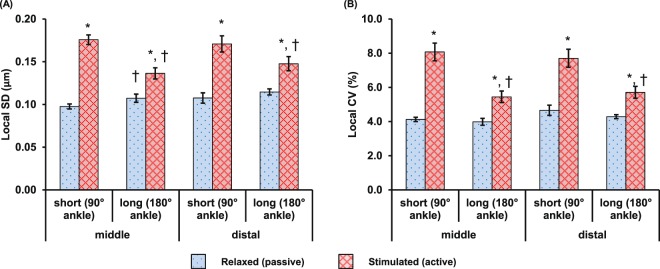
Activation-induced changes in local dispersion of sarcomere lengths in terms of (**A**) the local standard deviation (SD) and, (**B**) the local coefficient of variation (CV) measured at the mid- and distal TA when the TAs were set at the short and long lengths. The data were pooled from 9 animals for the mid-TA group and 8 animals for the distal TA group, respectively. The responses were similar for sarcomeres at the mid- and distal TA. Muscle activation led to increased dispersion in sarcomere lengths. The increase in dispersion was more pronounced for sarcomeres at the short compared to the long muscle length. *Indicates significant differences in local SD or local CV compared with sarcomeres in the relaxed muscle at either the short or the long length (*p* < 0.01). ^†^Indicates significant differences in local SD or local CV compared with sarcomeres in either the relaxed or the activated muscle at the short length (*p* < 0.01).

No statistical difference was found in the local SD or local CV of the SLs in the mid-TA and distal TA groups (Fig. 3). The baseline values of local CV in the passive muscle ranged between 4–4.5% at short and long lengths. Activation of the muscle caused a 33–96% increase in SL dispersion compared to the passive values. Increases in SL dispersion were 2–3 times greater at the short compared to the long muscle length.

**Figure 4:**
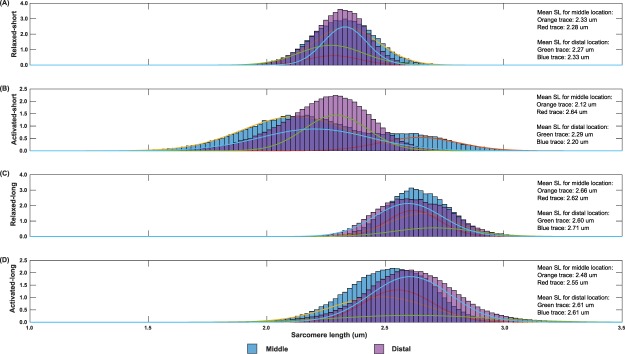
Probability distribution functions (PDFs) of sarcomere lengths pooled from 9 tested animals for the mid-TA (blue bars) and the 8 tested animals for the distal TA (purple bars) in the relaxed-short (**A**), activated-short (**B**), relaxed-long (**C**), and activated-long (**D**) muscles. Two weighted Gaussian curves (orange and red curves for the middle location; green and blue curves for the distal location) were fitted to the individual PDFs. In the relaxed muscles (**A**,**C**), the length distribution of sarcomeres at the middle and the distal locations was similar. In the activated-short muscle (**B**), the distribution of SLs at the mid-TA was biomodal with mean lengths of 2.12 µm and 2.64 µm, but the distribution of SLs at the distal TA was unimodal with a mean length of 2.25 µm. In the activated-long muscle (**D**), the length distribution of sarcomeres at the distal TA was shifted to the right of that at the mid-TA. Note that the area under the PDF and the total area under the two weighted Gaussian curves are, respectively, equal to 1.

The probability distribution function of SLs is shown in Fig. (4). SL distributions were wider in the activated muscles compared to those in the passive muscles, and were consistent with the results of SL dispersion presented in Fig. (3). The PDFs at the mid-TA and distal TA had a certain degree of overlap, except for the PDF at the distal TA in the activated-long state, which was shifted to the right of the PDF of SLs at the mid-TA (Fig. 4D). All but the SLs of the activated-short muscles in the mid-TA showed a normal, unimodal distribution. SLs in the mid-TA for the activated-short state had a bimodal distribution with mean lengths of 2.12 µm and 2.64 µm (Fig. 4B). Interestingly, these two mean lengths correspond to a theoretical force of 0.88 (arbitrary unit) despite residing, respectively, on the ascending limb and descending limb of the theoretical FL curve. The PDFs for individual animals (n = 9 for mid-TA; n = 8 for distal TA) are included in detail in the Supplementary Materials, S3 and S4.

**Figure 5:**
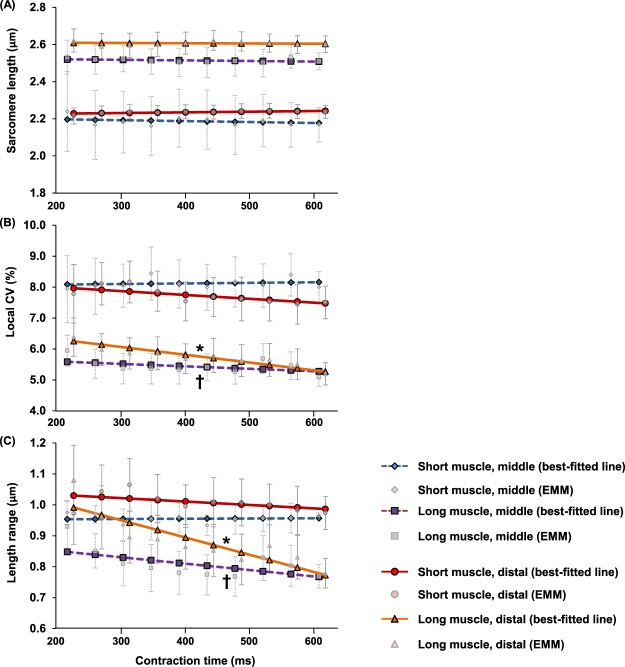
Progressive changes of (**A**) the mean sarcomere length (SLs), (**B**) the local coefficient of variation (CV) of SLs and, (**C**) the SL range, over the 600 ms contraction period measured at the mid- (dotted line) and distal (solid line) TA with the muscle being set at the short and the long lengths. For clarity, the data of the distal TA (solid line) was displaced 10 ms to the right of the data of the mid-TA (dotted line). The mean SLs at the mid- and distal locations stayed constant throughout the contraction period. For the short muscle, the local CV and the SL range did not change with the contraction time. At long muscle length, the local CV and the SL range at the mid- and distal TA decreased with contraction time. Specifically, the SL range at the mid-TA decreased from 0.85 µm to 0.77 µm while the SL range at the distal TA decreased from 1.00 µm to 0.77 µm at the end of the contraction period. *Indicates significant relationship between contraction time and local CV (or SL range) (*p* < 0.05). ^†^Indicates significant relationship between contraction time and local CV (or SL range) (*p* < 0.01). EMM- estimated marginal mean.

Over the 600 ms contraction period, the mean SLs at the mid- and distal TA stayed constant (Fig. 5A). The local CV and the SL range in the short muscle did not change with contraction time. However, the local CV and the SL range in the long muscle decreased with contraction time. Specifically, the SL range at the mid-TA decreased from 0.85 to 0.77 µm, while the SL range at the distal TA decreased from 1.0 to 0.77 µm during the contraction period (Fig. 5B,C).

**Figure 6:**
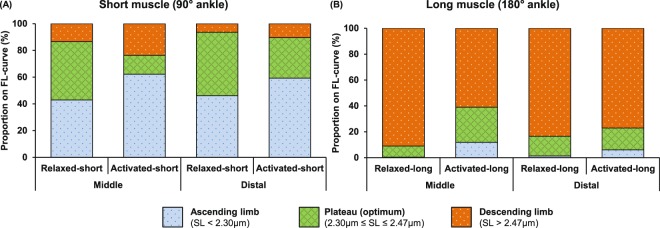
Proportion shift of sarcomeres at the mid- and distal TA with lengths that fall onto the ascending limb, plateau region (optimal length), and descending limb of the theoretical force-length curve due to muscle activation at short (**A**) and long (**B**) muscle lengths. At short muscle length, the proportion of sarcomeres with optimal lengths decreased during activation, with the decrease at the mid-TA being larger (~70%) than that at the distal TA (~35%). At the long muscle length, the activation resulted in an increase of the proportion of sarcomeres with optimal lengths at the mid-TA (from 10% to 25%), but did not change the corresponding proportion at the distal TA.

In the relaxed state, the proportion of sarcomeres residing on the ascending limb, plateau, and descending limb of the theoretical FL curve were similar for the mid-TA and distal TA sites (Fig. 6). The proportions shifted during muscle activation. At the short muscle length, the proportion of sarcomeres with optimal lengths decreased during activation, with the decrease at the mid-TA being larger (68%) than that at the distal TA (36%) (Fig. 6A). At long muscle length, activation resulted in more sarcomeres of the mid-TA being located at optimal lengths (from 9% to 27%), but did not change the proportion of sarcomeres on the plateau region for the distal TA (Fig. 6B).

**Figure 7:**
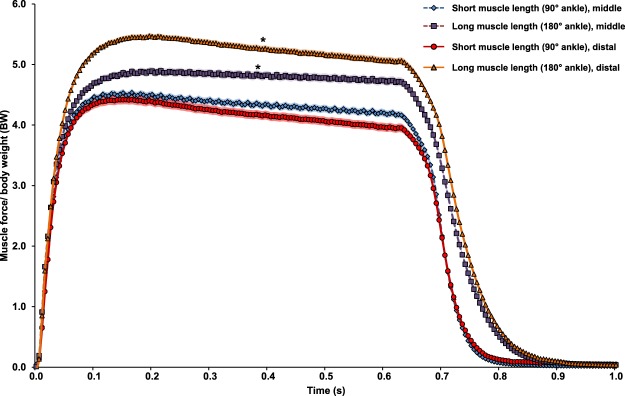
Mean muscle force normalized to body weight (BW) pooled from the trials conducted at the short (120 trials for mid-TA, and 169 trials for distal TA) and the long muscle lengths (174 trials for mid-TA and 159 trials for distal TA). The colour shading indicates ±1 standard error of the mean. On average, the TA muscle generated more force at the long (180° ankle flexion) than the short length (90° ankle flexion). There is no statistical difference between the mid-TA and distal TA groups. *Indicates significant differences in force compared with forces measured at the short muscle length in either the mid-TA or distal TA group (*p* < 0.01).

Finally, muscle forces generated at the long muscle length were 9–25% higher than those produced at the short muscle length (Fig. 7). There was no difference in muscle force between the mid-TA and distal TA groups.

## Discussion

**Figure 8:**
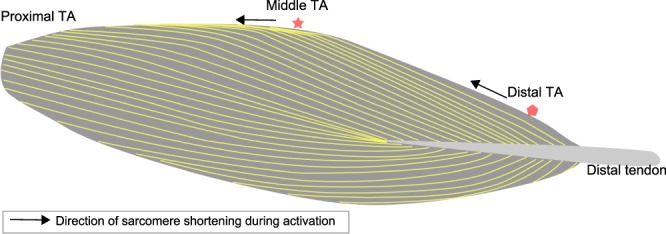
Schematic illustration of the muscle architecture in the mid-sagittal plane of a TA, with the fiber orientation being depicted by the yellow outlines^33,34^. The distal tendon lies in a horizontal plane. The forces generated by the muscle fibers at the mid-TA act in the horizontal plane, but the muscle fibers at the distal TA contract in a plane oblique to the horizontal plane.

The primary goal of this study was to compare the sarcomere lengths at two different locations in the intact and activated TA of living mice. The TA has a spindle-like shape, thus, the mid-TA has a bigger cross-sectional area and less surface tilt compared to the distal TA (Fig. 8)^33,34^. In a previous study, we found greater sarcomere elongations at the distal- compared to the mid-TA when the muscle was stretched passively^21^. Here, we wanted to see if SL dispersions became greater with activation, as observed in single myofibrils^11^, and if SL dispersions differed for different locations in a muscle. We found that while the sarcomere lengths at the mid- and distal TA were similar in the activated muscle, activation-induced shortening of the sarcomeres was not (Fig. 2A). During activation, sarcomeres at the mid-TA shortened by ~7%, while sarcomeres at the distal TA shortened by a mere 0–3%. It is not clear why sarcomere shortening upon muscle activation was different between the two sites, but differences in the elastic elements surrounding these sites may play a crucial role.

The SL dispersion had baseline values of ~4% CV in the passive muscle, and increased by 33–96% during activation. However, there was no difference in SL dispersion between the mid- and distal TA (Fig. 3). Interestingly, SL dispersions at the mid- and distal TA were more pronounced (2–3 times greater dispersions) at the short compared to the long TA length. This finding may be explained by the potential involvement of passive structural proteins, such as titin and collagen, that may stabilize sarcomeres better at long lengths when structural elements would be expected to be taut, compared to short lengths when structural elements might be slack^31,35,36^. Passive tension in mouse TA develops at SLs longer than ~2.5 µm^37^. For the passive experiments, SLs were ~2.3 µm and ~2.65 µm, respectively for the short and long TA experiments (Fig. 2). Therefore, it appears that at the long TA Length, passive structural proteins are engaged and provide stability, while at short TA lengths passive structural proteins may be slack and allow for greater SL dispersions. These intra-sarcomeric structural proteins may also be involved in stabilizing the SLs in the course of activation, as only the SLs in the long muscle showed a trend of increasing uniformity over the 600 ms isometric contraction period (Fig. 5B,C). Future studies are needed to investigate how sarcomeres are stabilized in entire muscles and why there are such great non-uniformities in SLs in passive and active muscle.

It should be pointed out that the highest CV found in the activated muscle was only 8%, which is 2.5–4 times lower than the CV reported in single myofibril experiments^11,13^. This implies that the structural proteins and the passive collagen fibril network present around the muscle fibers in the intact muscles have an attenuating effect on the SL non-uniformities. Despite the small SL dispersions in intact muscle (6–8%), the SL range in a small region of the muscle (159 × 2.8 µm^2^) easily reaches 1 µm for the activated TA (Fig. 2D). This difference in SL corresponds to more than 40% of the optimal sarcomere length in mouse skeletal muscle^5,38,39^. Also, a SL difference of 1 µm would theoretically correspond to a difference of 70% of the maximal isometric force if it occurred on the descending limb, and would be the difference between zero force and maximal force if it happened on the ascending limb. However, the functional implication of the SL non-uniformity remains unclear, but future studies need to investigate what measure of SLs (e.g., mean, median, percent sarcomeres on the plateau region, SL range or CV of SL) may be most appropriate when attempting to predict even the simplest functional properties, such as the maximal isometric force, from some measures of sarcomere length. At present, it has been implicitly assumed that a mean measurement of SLs, as obtained for example by laser diffraction, at a single muscle lengths and in the passive state, provides a good estimate of the maximal isometric force^8,29,40,41^. However, studies testing this idea explicitly have not been performed. Also, studies measuring SLs and muscle force in entire muscles are rare. But even if they have been done, the optimal sarcomere lengths were found to be vastly different from those predicted theoretically for optimal lengths. For example, Rack and Westbury^40^ showed maximal, active isometric forces for cat soleus at a mean sarcomere length of about 3.0 µm (mean SL measured at one muscle length and for passive conditions, see their Fig. 3), while the theoretical optimal sarcomere lengths for cats, based on direct actin and myosin lengths measurements, is between 2.34 and 2.51 µm^5^. This rare example where mean SL and force have been measured in an entire muscle illustrates that current assumptions of SLs and mechanical properties for entire, *in vivo* skeletal muscles should be viewed with caution.

We also found that the distribution of SLs on the theoretical FL curve was different between the mid- and distal TA. In particular, we are interested in these differences in the active TA, as we showed previously that the SLs in the active TA are more reliable predictors of isometric muscle force than the corresponding passive SLs^25^. By comparing the SL distribution in the active TA between the short and long length conditions, we found that a smaller percentage of sarcomeres (14%) were at optimal length for the short than the long TA (27%) for the mid-TA location. The opposite result was obtained for the distal TA location where the percentage of sarcomeres residing on the plateau region of the FL curve was greater at the short (30%) compared to the long TA length (17%). Due to the spindle-like shape of the TA, the force generated by individual muscle fibers, and therefore the contractile behaviour of the corresponding sarcomeres, may depend on the orientation of the muscle fibers relative to the distal tendon. Indeed, sarcomeres in the mid-TA shortened in a plane parallel to the distal tendon, while sarcomeres in the distal TA shortened in a plane oblique to the distal tendon (Fig. 8). Therefore, the local structure and geometry of a muscle may cause the differences in SL distribution measured at the mid- and distal TA locations.

There are limitations in this study that need to be taken into account when interpreting our results. First, multiple layers of skin and fascia overlying the TA were removed for SHG imaging, but the epimysium was left intact. Since the gross structure of the TA was unperturbed (Fig. 1), we assumed that the sarcomere lengths measured in this manner were identical to those that would have been obtained in the intact muscle embedded in all its connective tissue layers. Second, sarcomere images were obtained from the top ~100 µm of the muscle only, as images deeper in the muscle are of poor quality in terms of signal-to-noise ratio. Therefore, the SL results presented here may not be representative of deeper parts of the TA. Third, the sarcomere lengths measured in the current study were derived from the distance between adjacent A-band centers instead of the structural definition of a sarcomere that is bounded by two adjacent Z-lines. This may result in an overestimation of sarcomere length non-uniformities, especially in the case of A-band shifts^14^. However, significant A-band shifts only seem to occur in prolonged muscle activation that lasts for tens of seconds (>100 s in Horowits and Podolsky^35^). For our 600 ms contractions, we would expect A-band shifts to be ~0.02 µm at most^42^, which would be negligible considering that we observed length differences in neighbouring sarcomeres reaching as high as 1.0 µm (Fig. 2D). Fourth, the buckle force transducer sat on the tibia during the implantation onto the distal TA tendon, and such bone impingement may lead to an underestimation of the muscle force. However, the backbone of the E-shaped transducer was free to rotate during the muscle contraction. This allows the middle arm of the transducer to deflect freely in response to the muscle force (see Supplementary Material, S5, for details), thus minimizing the bone impingement artifacts. Furthermore, every attempt was made to maintain the force transducer in the same position for testing and calibration, thereby ensuring that the resulting calibration curve between transducer voltage output and actual force was the same as that obtained during muscle testing. Finally, the SHG images acquired in the relaxed and activated states represent sarcomeres that are within an area of ~50 µm in diameter in the muscle, and not sarcomeres within single myofibrils.

Despite the aforementioned limitations, our study provides unique and novel data on sarcomere lengths at different locations in an intact muscle with distinctly different geometries and surface orientation, and for passive and active conditions. Future studies should focus on studying the functional implications of the attenuated sarcomere non-uniformity in intact muscles compared to isolated muscle fibers and myofibrils. As the sarcomere lengths vary across a whole muscle^21,30^, future investigations should also study sarcomeres at the proximal, medial and lateral TA to give a complete picture of the contractile behaviours of sarcomeres in a muscle. Finally, with the common use of mean sarcomere lengths from a single muscle length under passive conditions as a predictor variable of mechanical properties of muscles, and as an input into theoretical models of musculoskeletal systems, a systematic validation of this practice is urgently needed.

## Conclusion

Based on the results found in this study, we conclude that: (i) sarcomere length dispersions in the mouse TA increase substantially from the passive to the active state, but there was no difference in SL dispersion between the mid- and distal TA; (ii) SL non-uniformities within a small segment of muscle comprised of approximately 30 sarcomeres can easily reach 1.0 µm, which can mean that one sarcomere can be at optimal length and a neighbouring one at a length where it cannot produce active force. We would like to propose that providing mean sarcomere lengths from a single passive muscle length as an estimate of muscle mechanical properties, such as the force-length relationship, may be erroneous. Systematic studies on how to interpret muscle mechanical properties with measures of SL need to be performed to provide understanding in this research area.

## Electronic supplementary material

Supplementary Information

## Data Availability

All data that support the findings of this study are available from the corresponding author upon reasonable request.
