# Supplementary material for: Single sarcomere contraction dynamics in a whole muscle
Source: Sci Rep. 2018 Oct 15;8:15235. doi: 10.1038/s41598-018-33658-7 (PMC6189036; doi:10.1038/s41598-018-33658-7)
Supplement: Supplementary file 1 — Supplementary Information [file 41598_2018_33658_MOESM1_ESM.docx]

**Single sarcomere contraction dynamics in a whole muscle**

Eng Kuan Moo^1^, Walter Herzog^1^

^1^Human Performance Laboratory, Faculty of Kinesiology, University of Calgary, Calgary, Alberta, Canada

*S1: Determination of the scanning regions in the muscle under relaxed and activated conditions*

In order to estimate the total displacement of the site of sarcomere length measurement during muscle activation, two fluorescent markers separated by ~1mm along the muscle longitudinal axis were attached to the mid-belly of the muscle by using a 100µm-diameter glass tip attached to a 3-axes linear micro-manipulator (Newport Corp., CA, USA). The displacements of these two fluorescent markers (*d_1_*, *d_2_*, see Fig. R3) were carefully measured by observing through the eyepiece of a multi-photon excitation microscope (FVMPE-RS model, Olympus, Tokyo, Japan) under fluorescent light.

A scanning area was selected between the two fluorescent markers and the TA muscle was imaged in its relaxed state. The displacement of the scanning area (*d_scanning_area_*) during muscle activation was determined by linear interpolation of the displacements of the two fluorescent markers (*d_1_*, *d_2_*). A sub-micron precision, motorized microscope stage (Prior Scientific, MA, USA) on which the animal sat was then used to displace the TA muscle by ‘*d_scanning_area_*’ in the horizontal plane (XY-plane). The TA muscle was then supra-maximally stimulated and imaged simultaneously at this XY-coordinate in order to image sarcomeres from the same region (within an area of ~50µm diameter) as in the relaxed muscle.


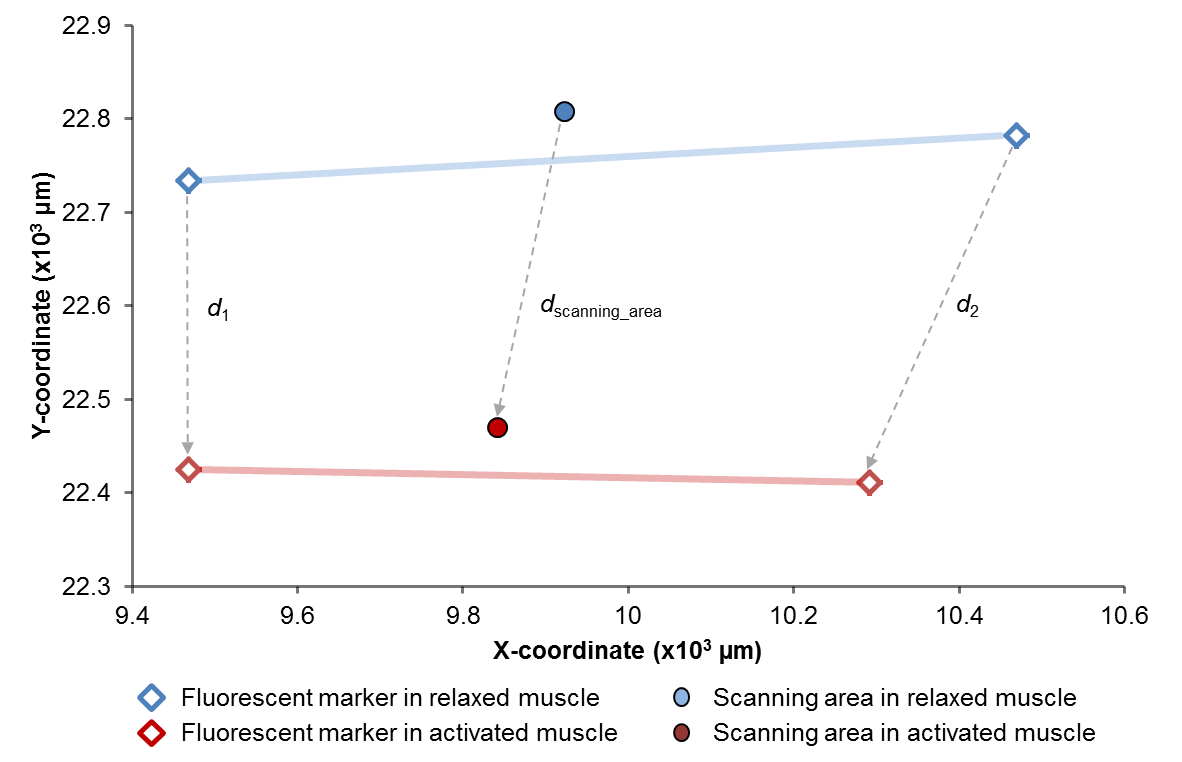


Fig. R1. XY-coordinate map on a sub-micron precision motorized microscope stage showing the displacements of the fluorescent markers (diamonds in blue and red) on the mouse TA during activation. The displacements of these two fluorescent markers were then used to determine the displacement of the scanning area (circles in blue and red) during activation to allow for imaging of sarcomeres from the same location in the relaxed and active state.

*S2: Correction for out-of-plane orientation in sarcomere length (SL) measurements*

Using the through-thickness muscle image in the XZ-plane (Fig. R4A), the orientation of the epimysium (*θ_surface_*) and the angle between the epimysium and the sarcomeric A-bands (*θ_A-band_*) were determined (Fig. R4B). As the time-series planar images of sarcomeres were taken in the horizontal plane, the SL measured (*SL_measured_*) from this images were corrected for out-of-plane projection using the sine rule as shown in Eq. (R1) and Eq. (R2) to obtain SL along the epimysium (*SL_corrected_*):

$\frac{{SL}_{corrected}}{\sin(180- \theta_{surface}-\theta_{A-band})}=\frac{{SL}_{measured}}{\sin(\theta_{A-band)}}$ (R1)

${SL}_{corrected}=\frac{\sin\left( 180- \theta_{surface}-\theta_{A-band} \right)}{\sin(\theta_{A-band)}}{\times SL}_{measured}$ (R2)


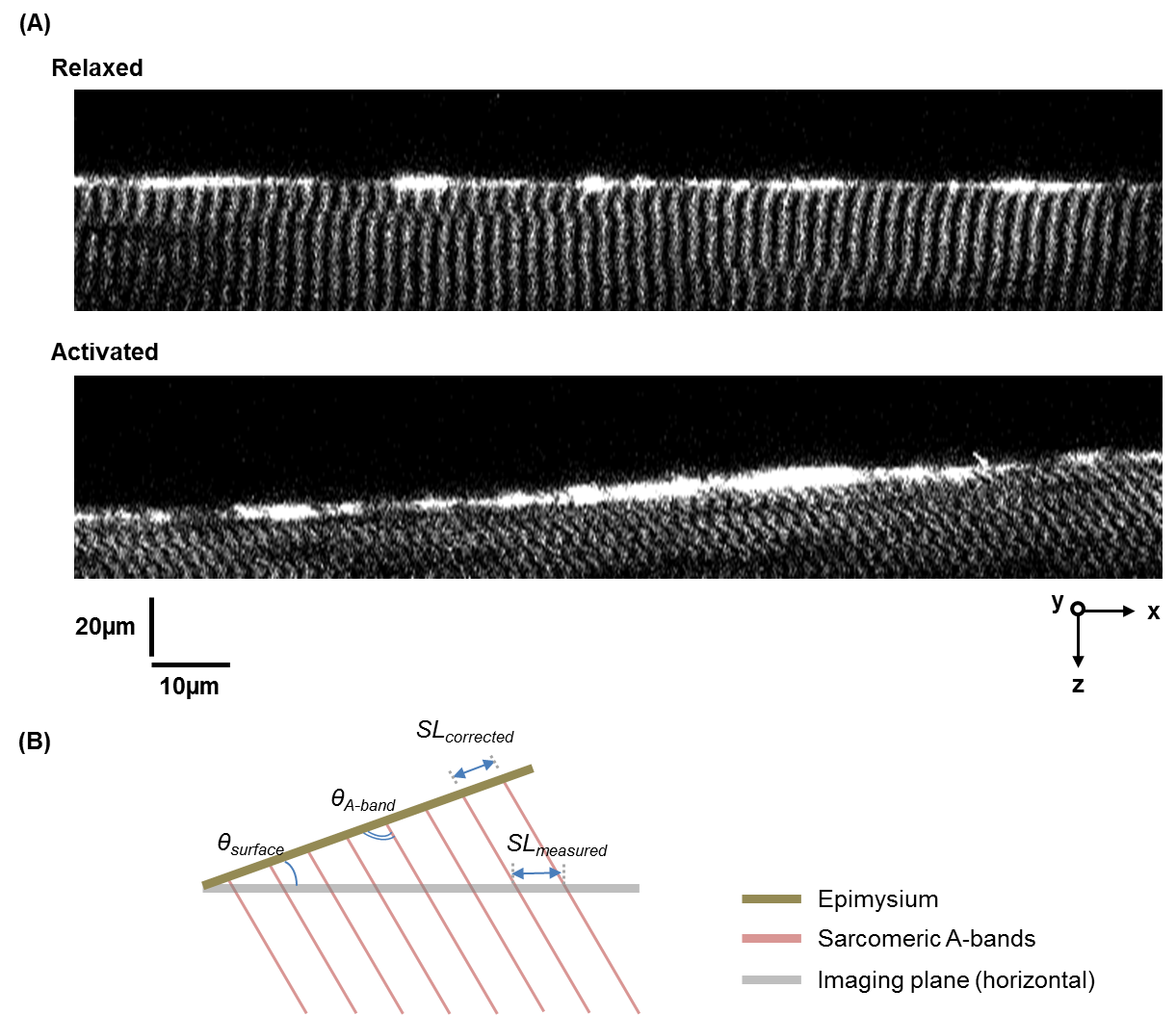


Fig. R2. (A) Through-thickness muscle taken in the XZ-plane showing the orientations of the epimysium (top layer) and the sarcomeric A-bands (white bands) in the relaxed and activated muscle. (B) Schematic diagram showing the angles (*θ_surface_* and *θ_A-band_*) measured from the through-thickness images in order to correct for the out-of-plane projection and obtain sarcomere lengths along the epimysium (*SL_corrected_*).

*S3: (SL) probability distribution functions (PDF) measured at the mid-TA in individual animals (n=9)*

Animal ID: 1


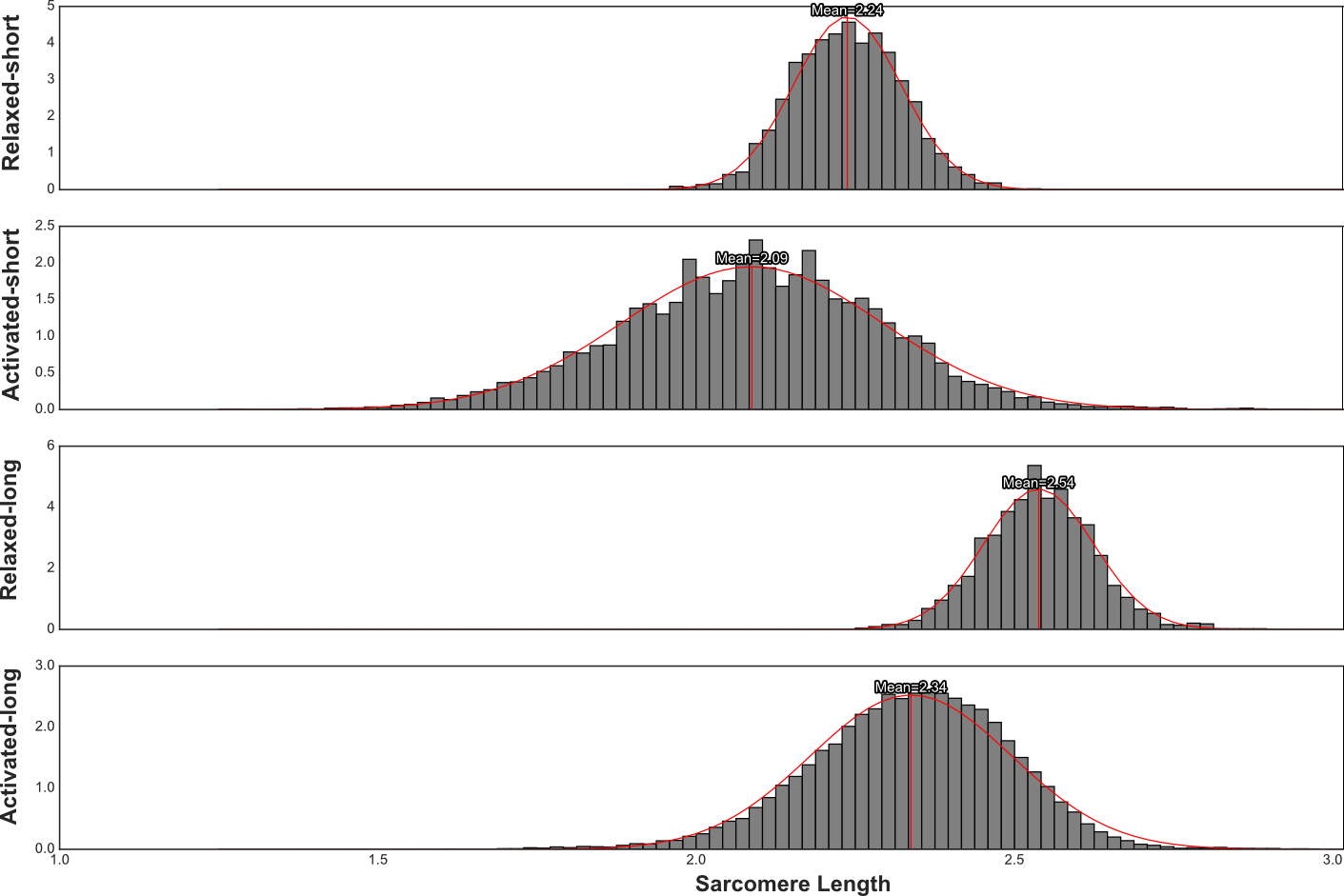


Fig. R3. SL PDF measured at short and long muscle lengths under relaxed and activated conditions at mid-TA for ‘animal 1’.

Animal ID: 2


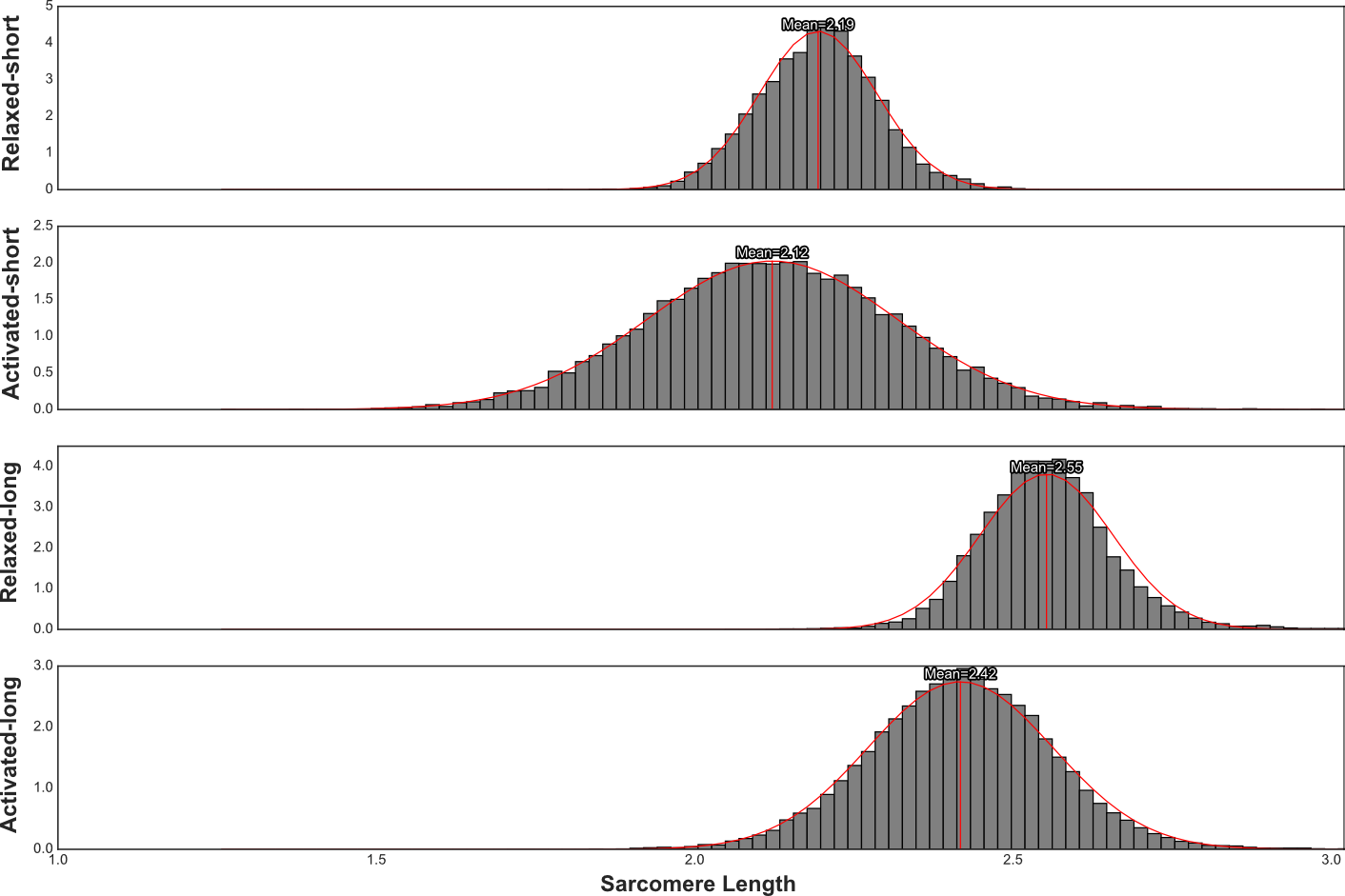


Fig. R4. SL PDF measured at short and long muscle lengths under relaxed and activated conditions at mid-TA for ‘animal 2’.

Animal ID: 3


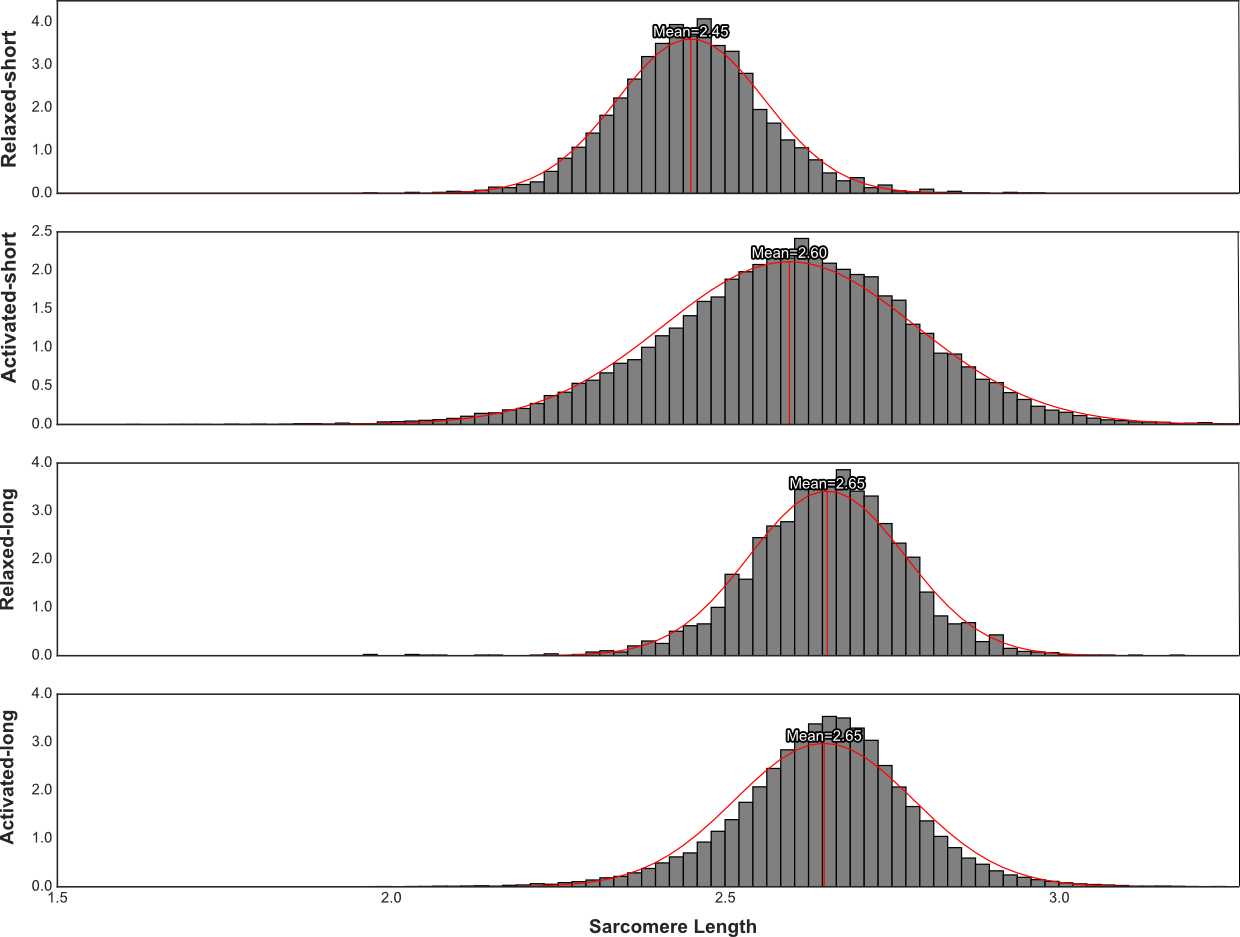


Fig. R5. SL PDF measured at short and long muscle lengths under relaxed and activated conditions at mid-TA for ‘animal 3’.

Animal ID: 4


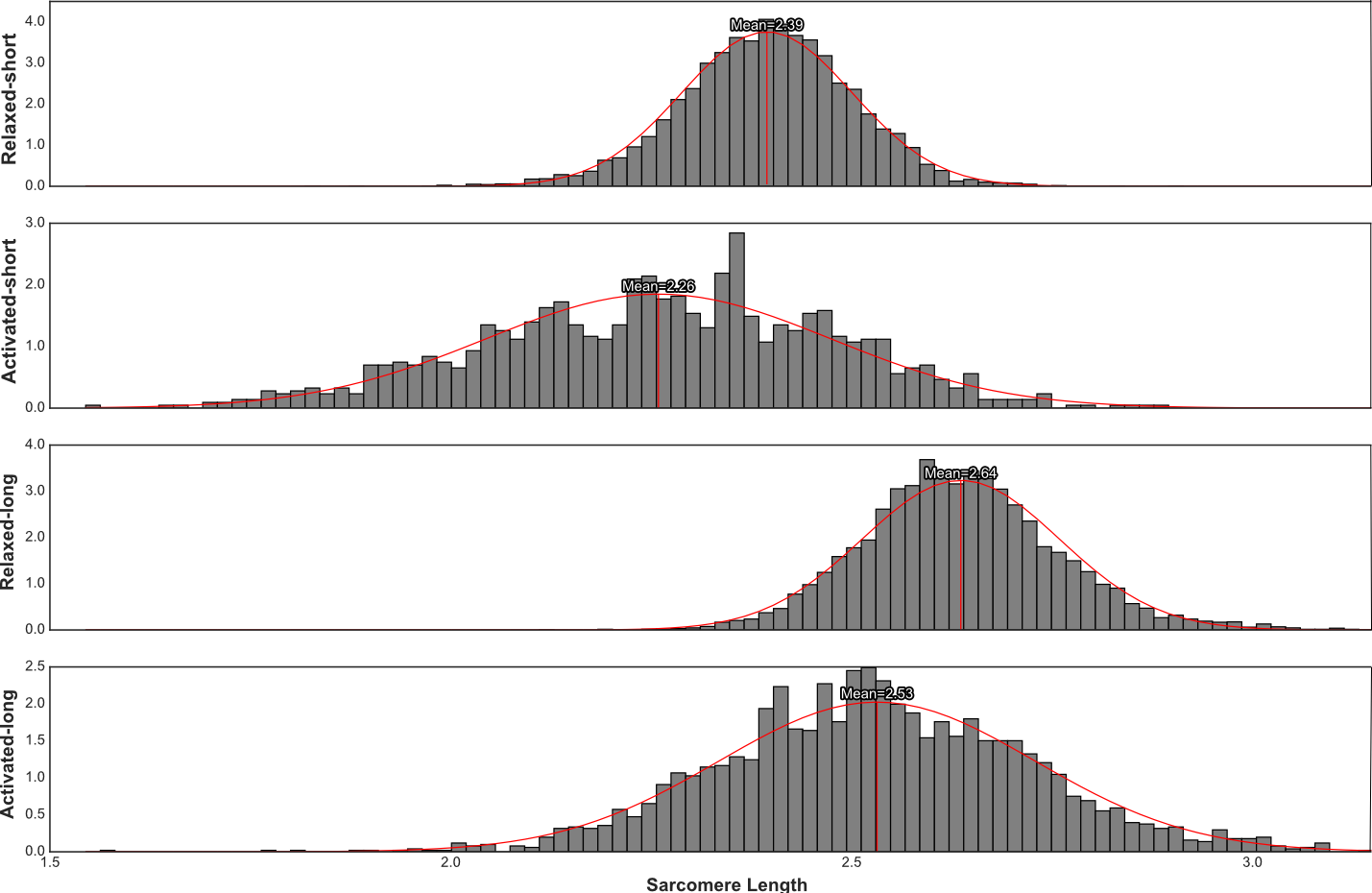


Fig. R6. SL PDF measured at short and long muscle lengths under relaxed and activated conditions at mid-TA for ‘animal 4’.

Animal ID: 5


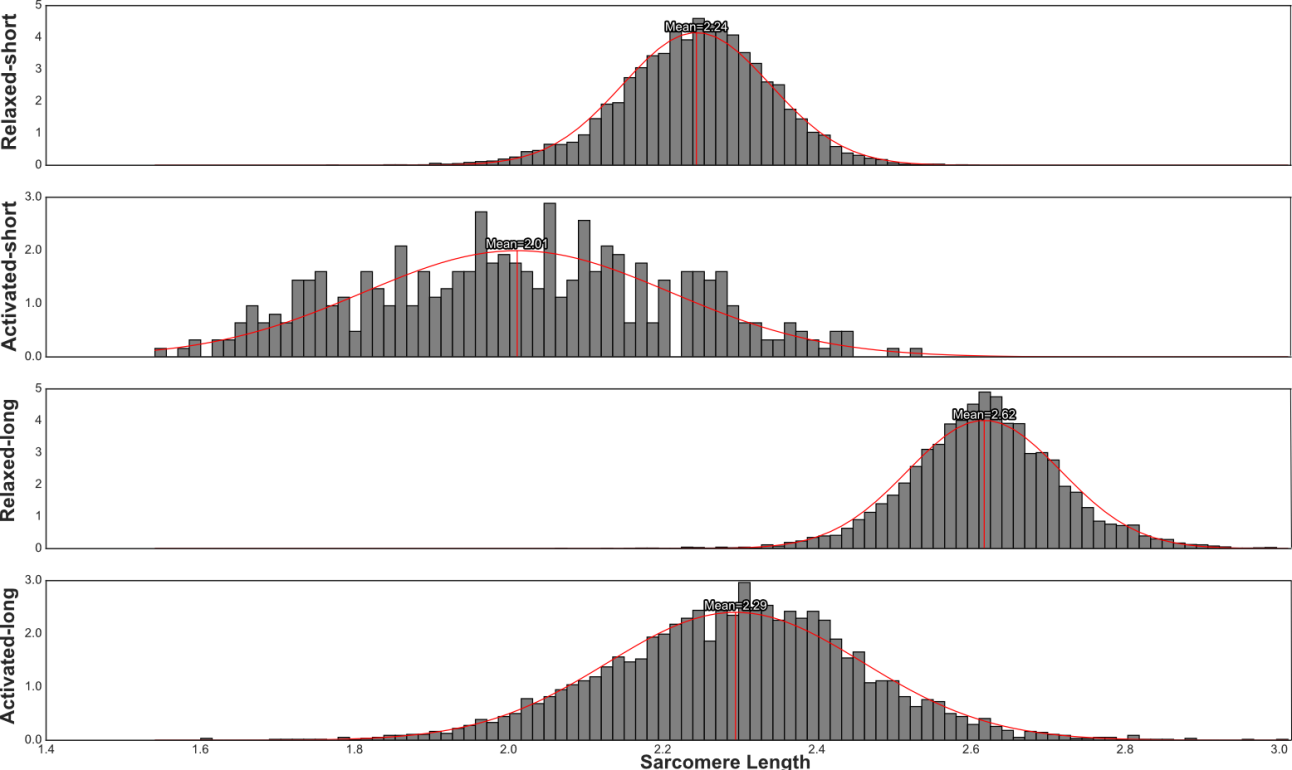


Fig. R7. SL PDF measured at short and long muscle lengths under relaxed and activated conditions at mid-TA for ‘animal 5’.

Animal ID: 6


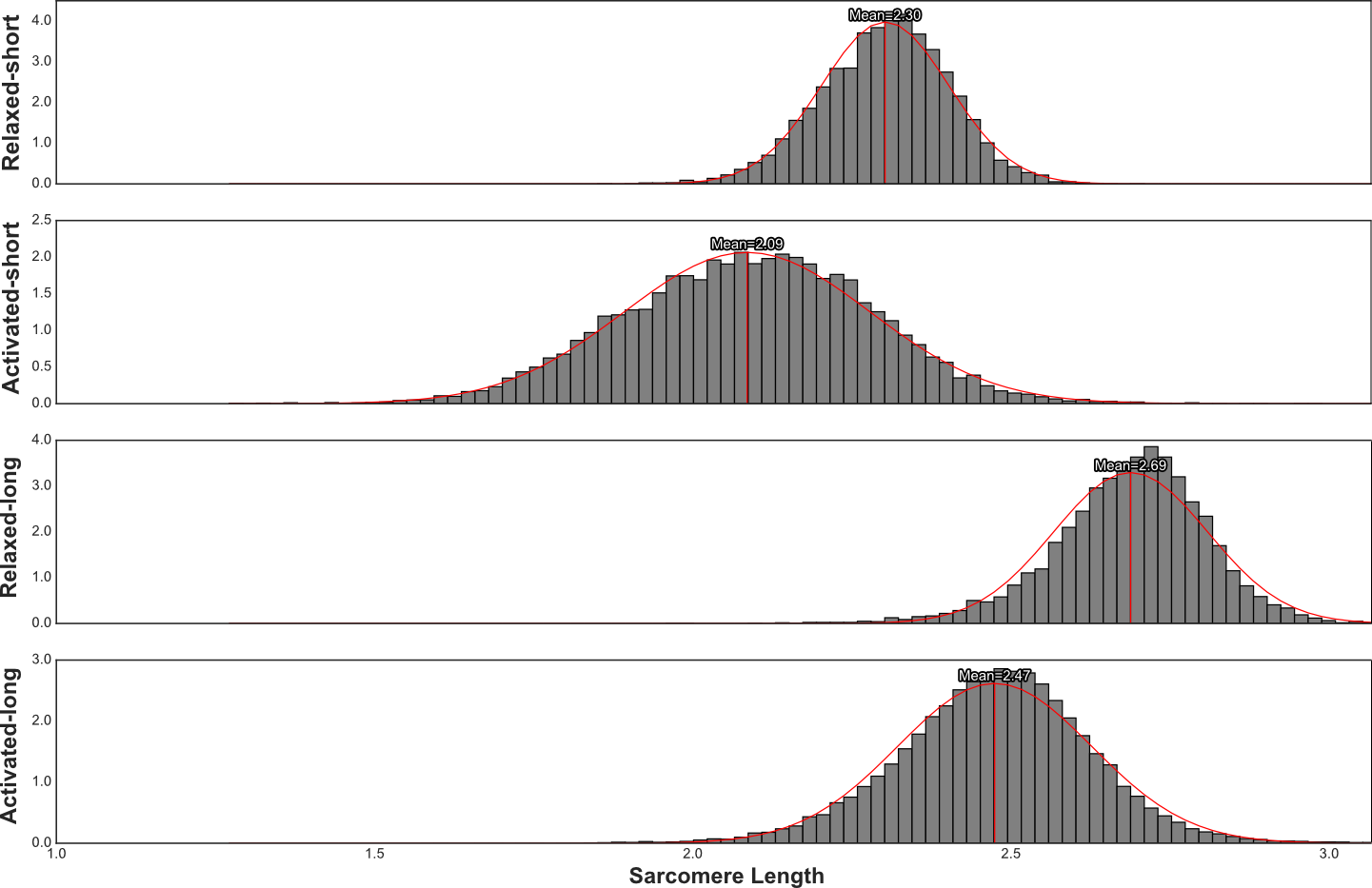


Fig. R8. SL PDF measured at short and long muscle lengths under relaxed and activated conditions at mid-TA for ‘animal 6’.

Animal ID: 7


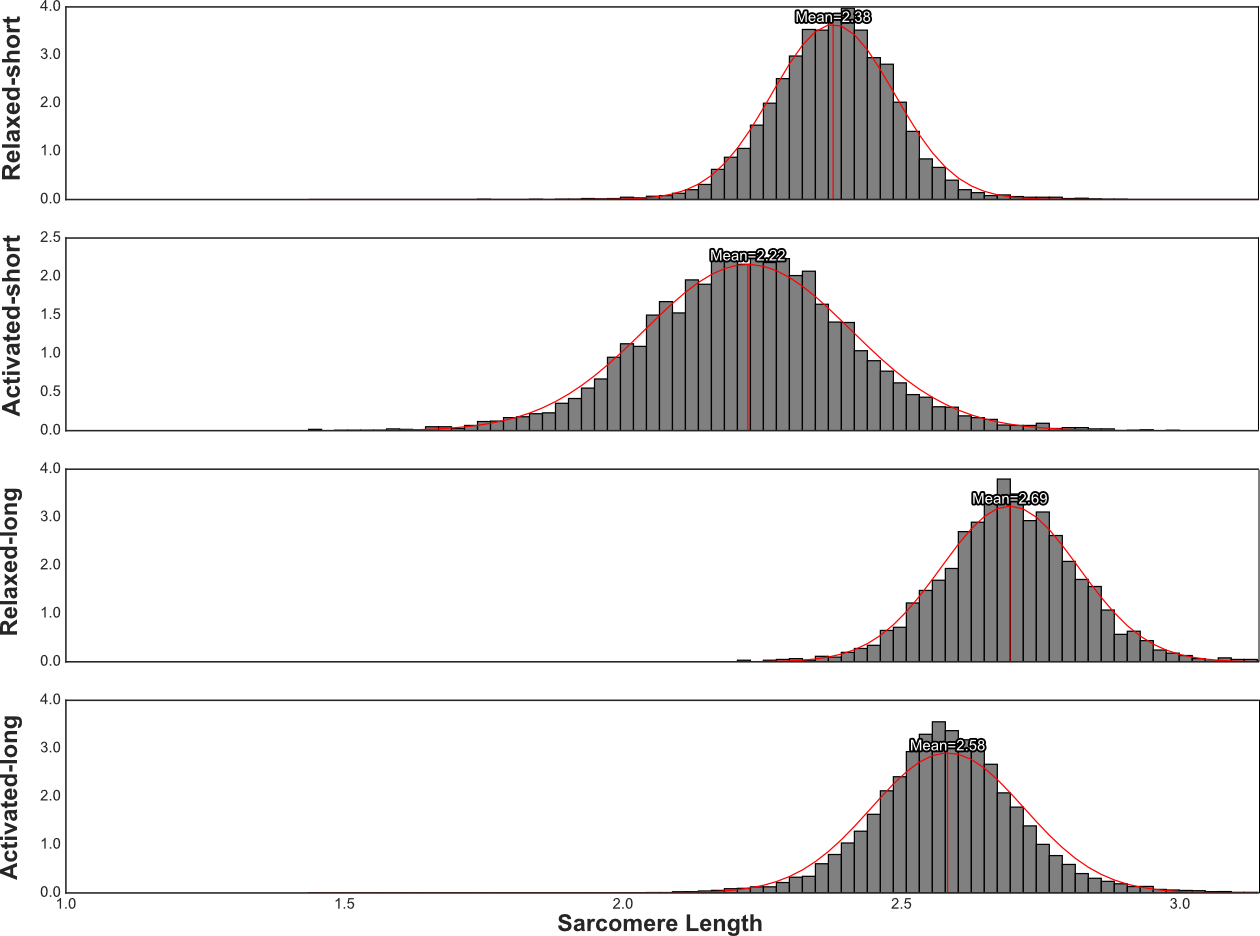


Fig. R9. SL PDF measured at short and long muscle lengths under relaxed and activated conditions at mid-TA for ‘animal 7’.

Animal ID: 8


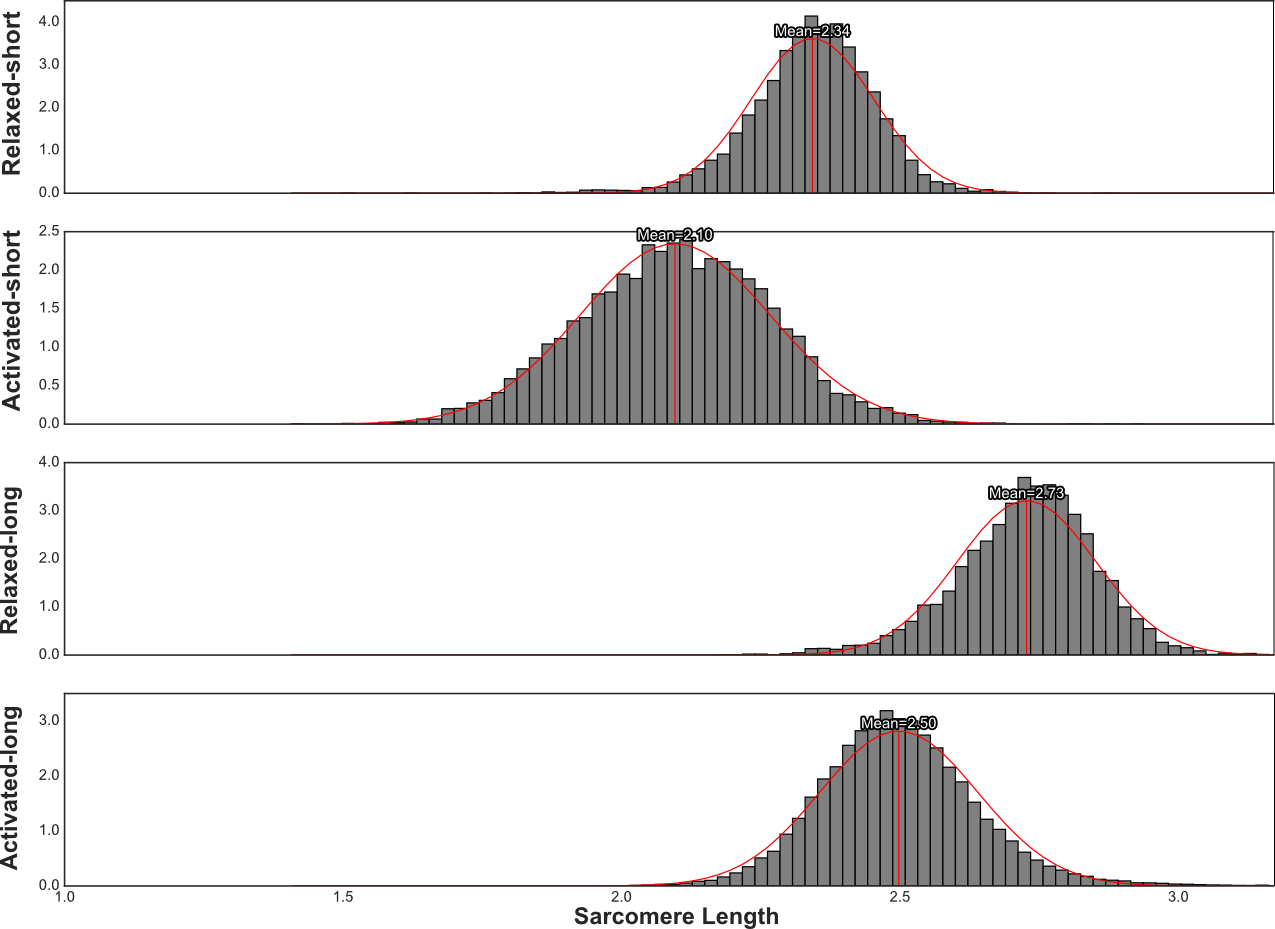


Fig. R10. SL PDF measured at short and long muscle lengths under relaxed and activated conditions at mid-TA for ‘animal 8’.

Animal ID: 9


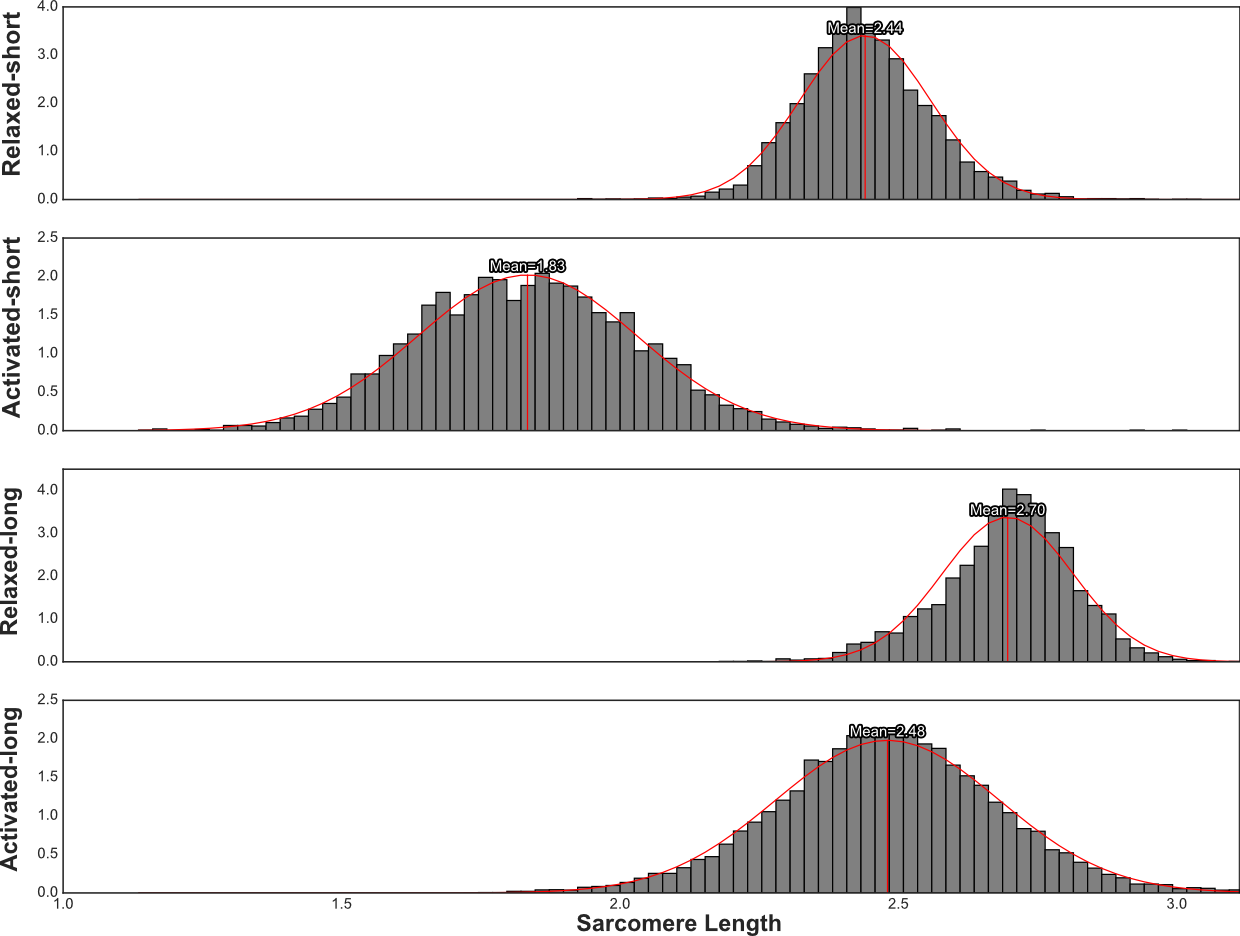


Fig. R11. SL PDF measured at short and long muscle lengths under relaxed and activated conditions at mid-TA for ‘animal 9’.

*S4: (SL) probability distribution functions (PDF) measured at the distal TA in individual animals (n=8)*

Animal ID: 10


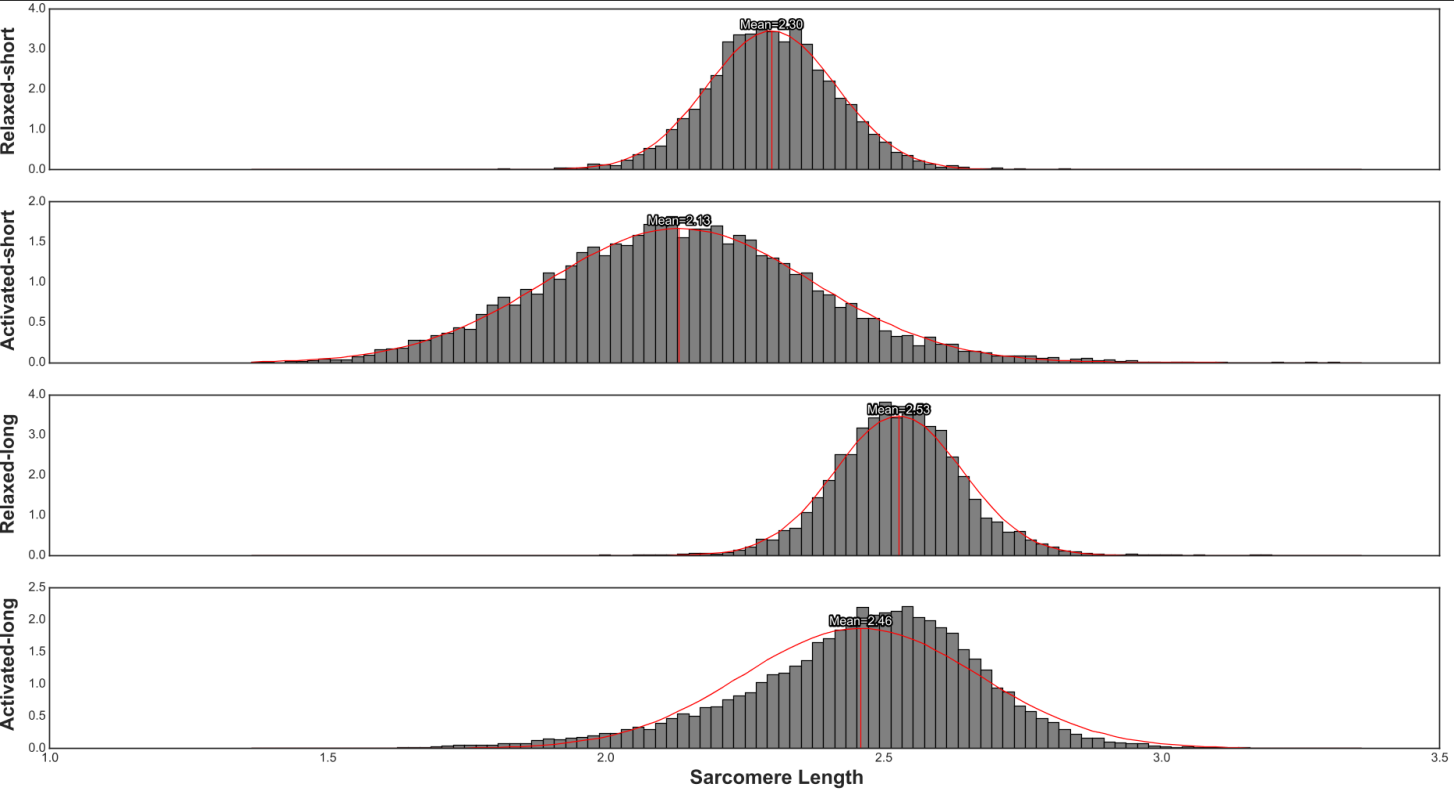


Fig. R12. SL PDF measured at short and long muscle lengths under relaxed and activated conditions at distal TA for ‘animal 10’.

Animal ID: 11


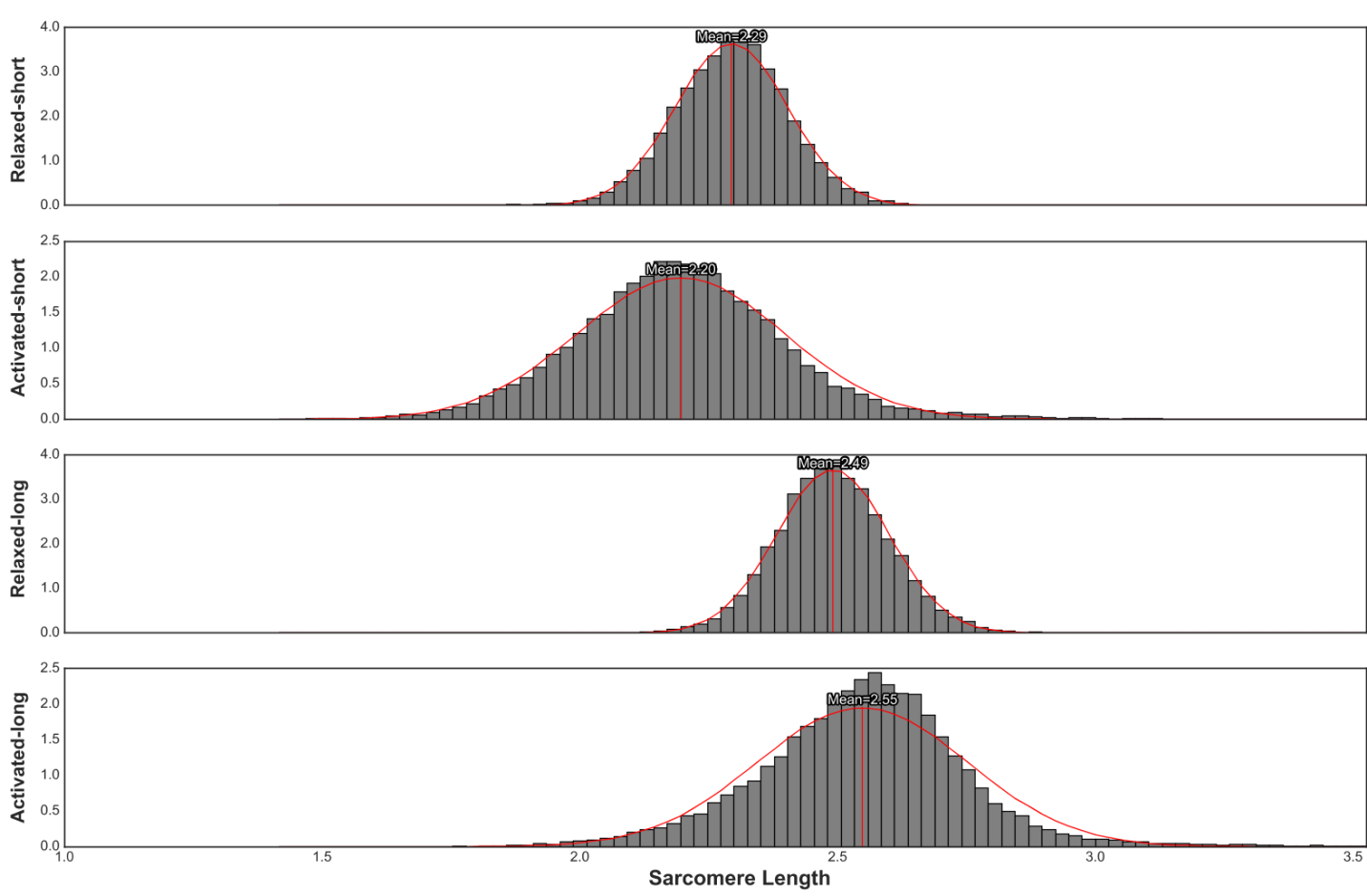


Fig. R13. SL PDF measured at short and long muscle lengths under relaxed and activated conditions at distal TA for ‘animal 11’.

Animal ID: 12


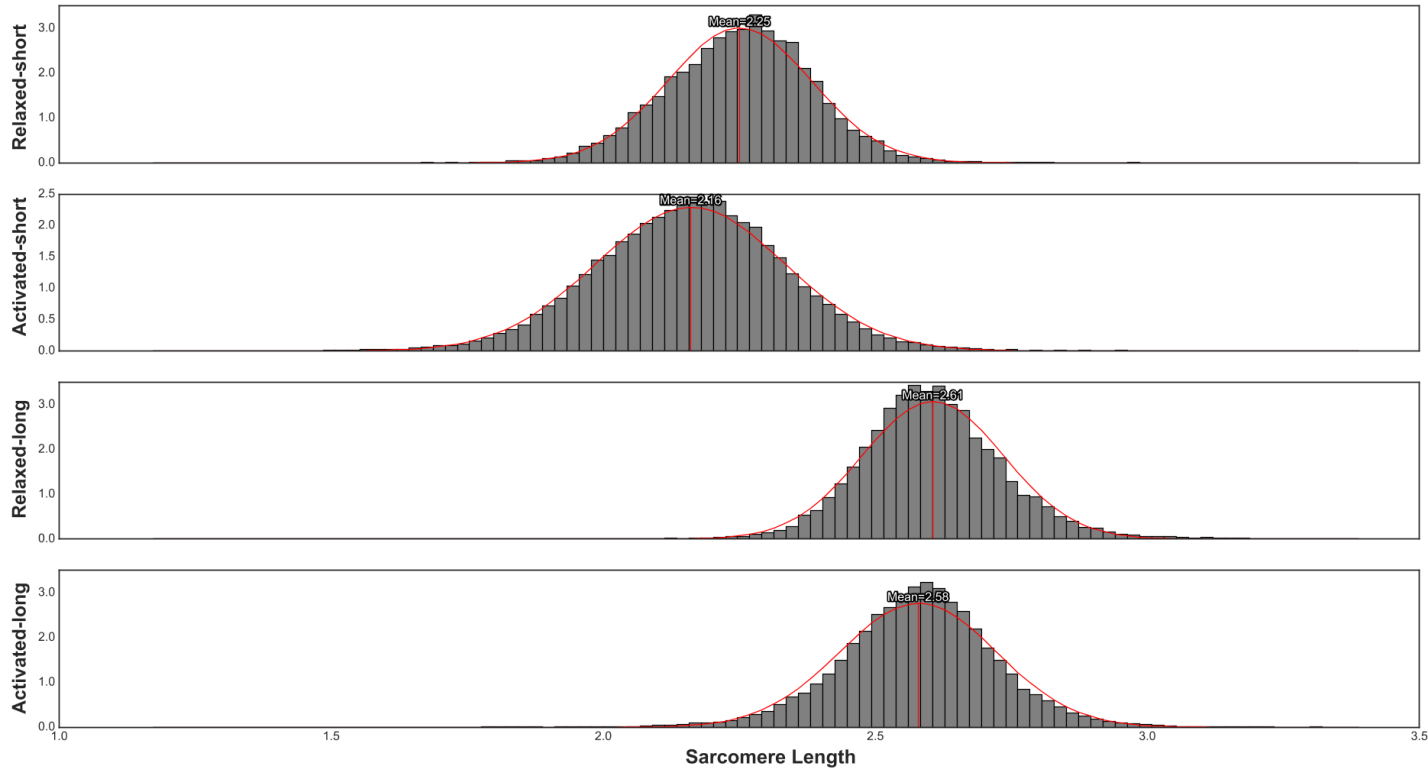


Fig. R14. SL PDF measured at short and long muscle lengths under relaxed and activated conditions at distal TA for ‘animal 12’.

Animal ID: 13


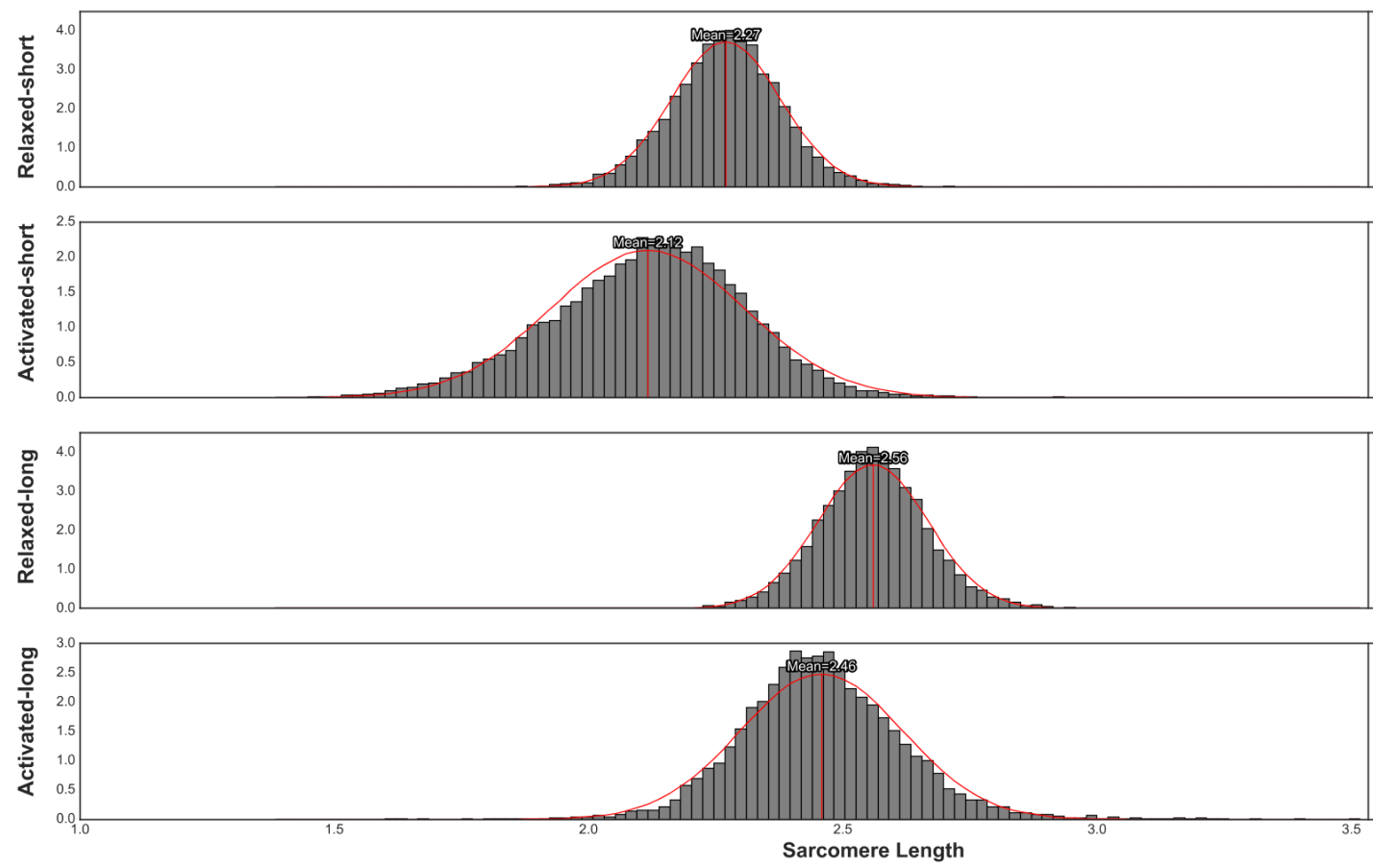


Fig. R15. SL PDF measured at short and long muscle lengths under relaxed and activated conditions at distal TA for ‘animal 13’.

Animal ID: 14


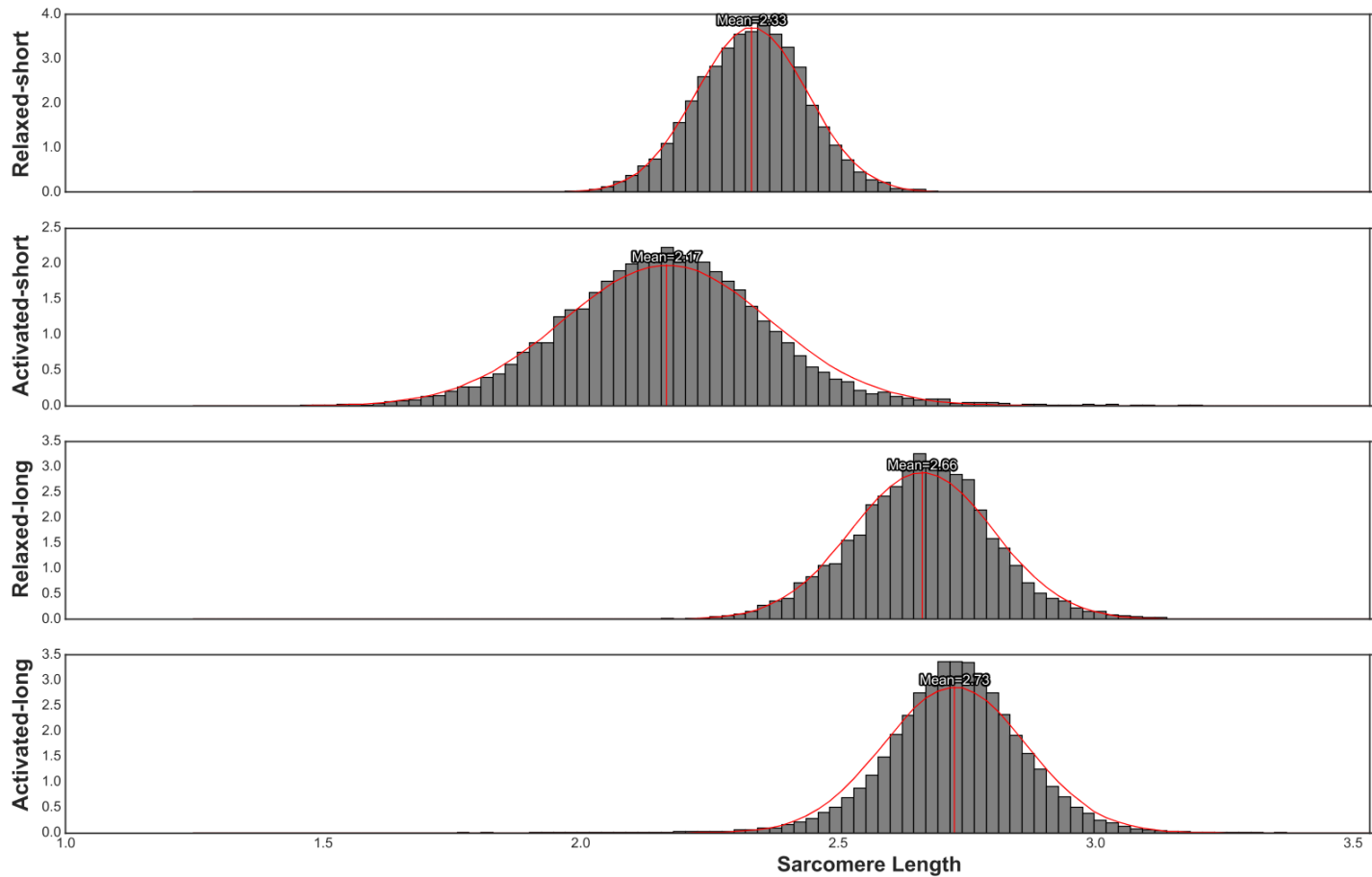


Fig. R16. SL PDF measured at short and long muscle lengths under relaxed and activated conditions at distal TA for ‘animal 14’.

Animal ID: 15


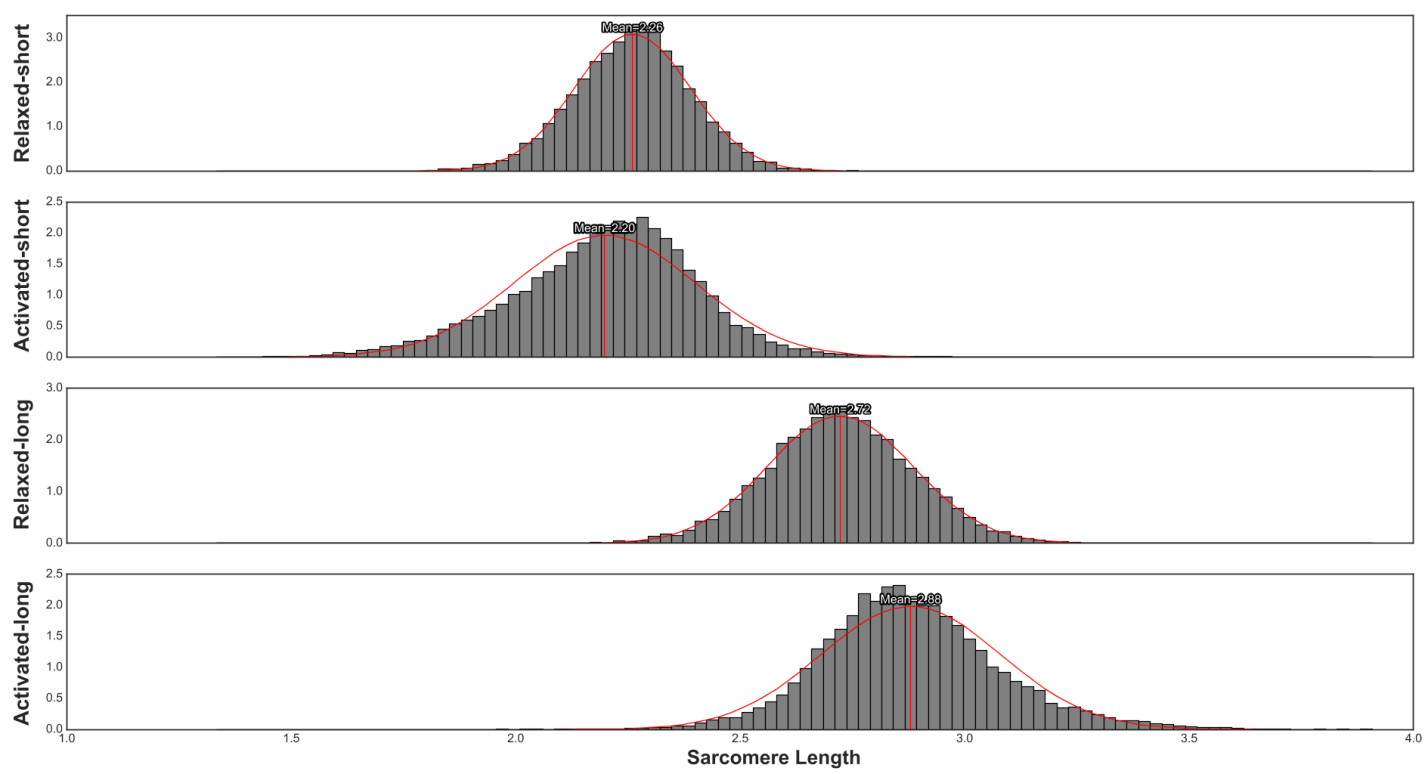


Fig. R17. SL PDF measured at short and long muscle lengths under relaxed and activated conditions at distal TA for ‘animal 15’.

Animal ID: 16


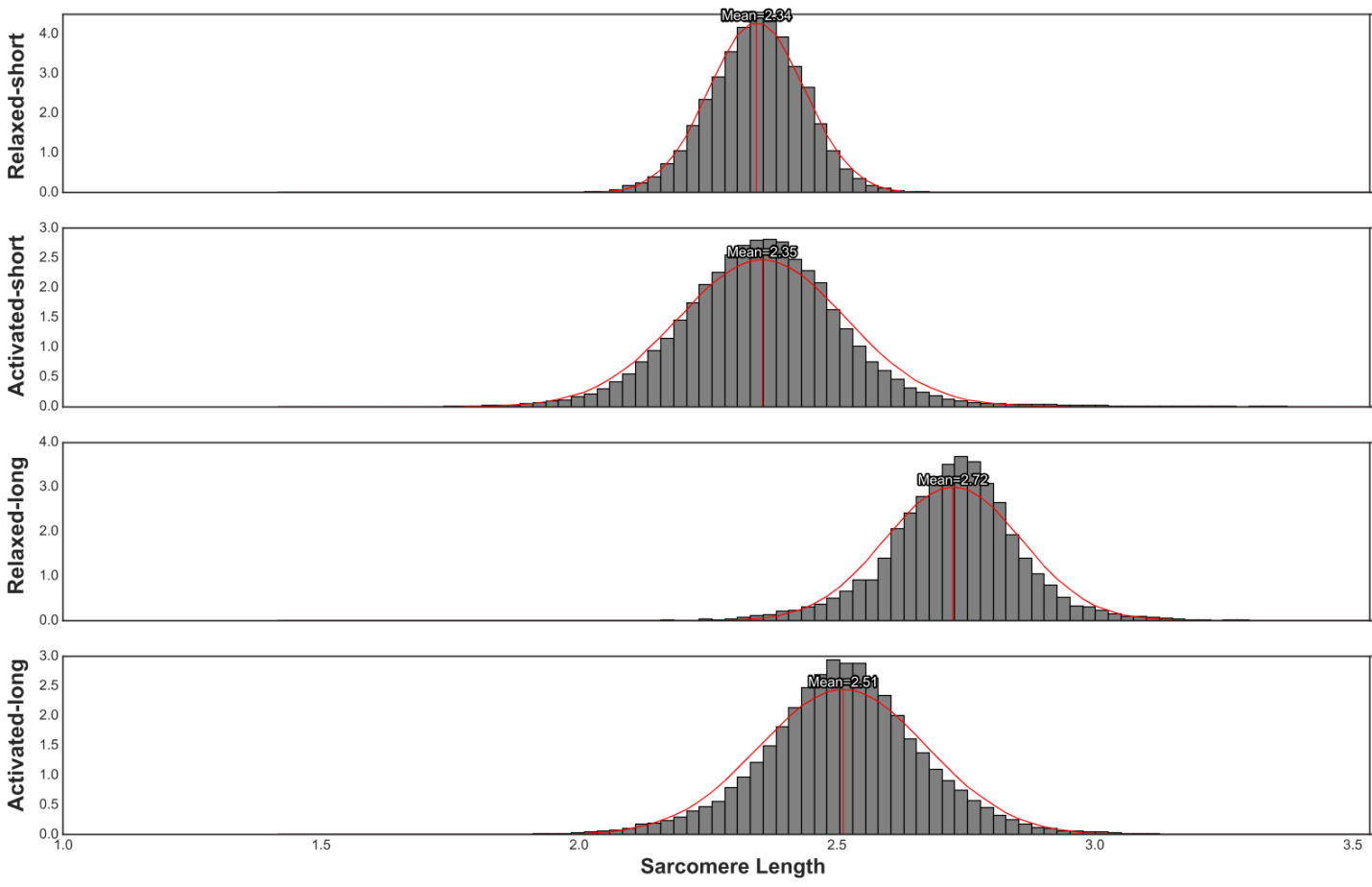


Fig. R18. SL PDF measured at short and long muscle lengths under relaxed and activated conditions at distal TA for ‘animal 16’.

Animal ID: 17


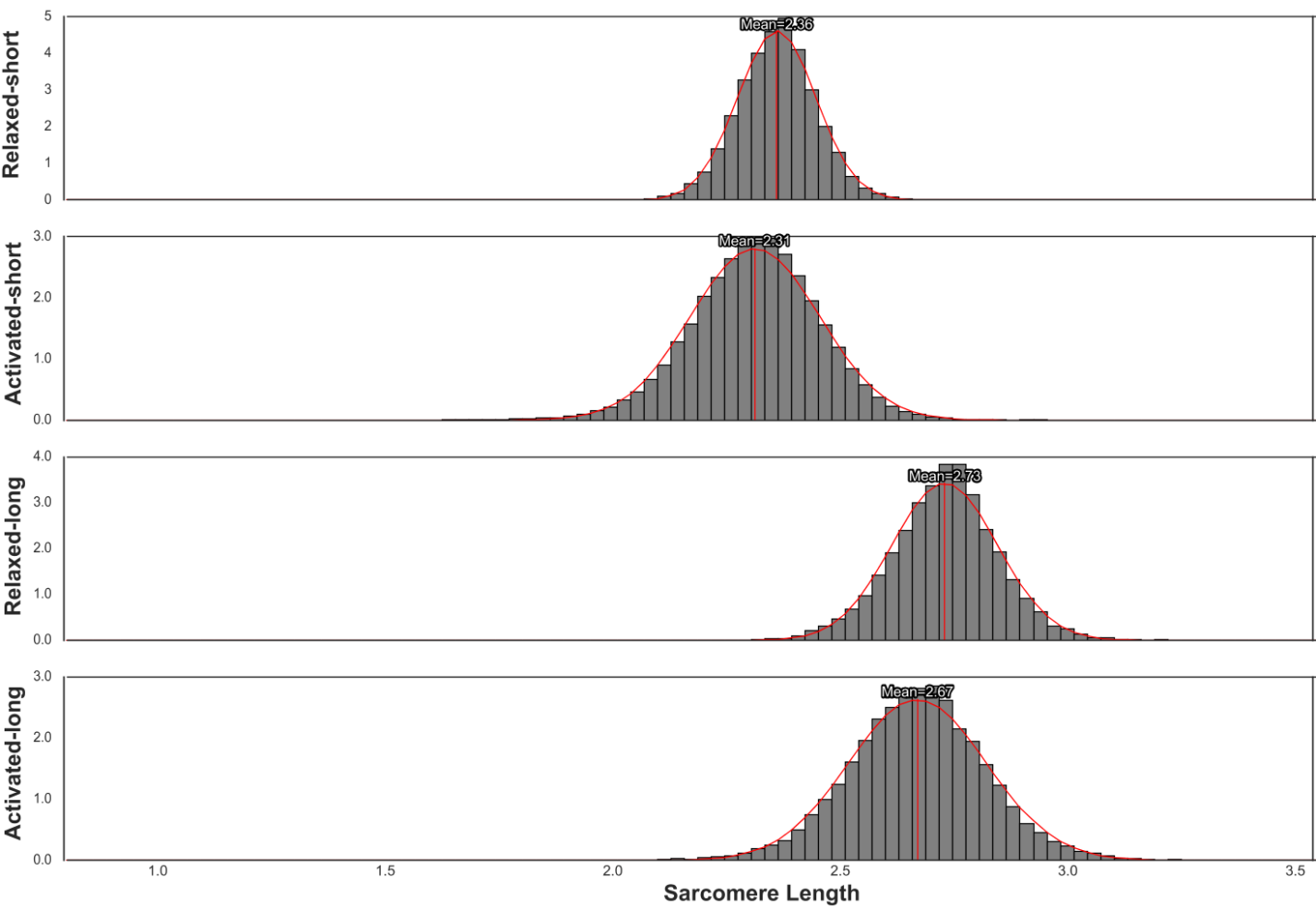


Fig. R19. SL PDF measured at short and long muscle lengths under relaxed and activated conditions at distal TA for ‘animal 17’.

*S5: Buckle-type force transducer*

The E-shaped buckle transducer used is 3.6mm in length (Fig R20). The force transducer was implanted onto the distal tendon of the TA and placed superior to the retinaculum (Fig R21), which was sufficient for accommodating the transducer. The open ends of the E were closed by sutures to secure the tendon at fixed position during muscle contraction. The arms of the E-shaped transducer sat on the tibia, the backbone of the transducer was unrestricted and was free to rotate during muscle contraction, thereby allowing the middle arm of the E-shaped transducer to deflect freely in response to the muscle force (Fig. R21). Care was taken during the calibration experiments to ensure the tendon force transducer to stay in the same position as during the muscle activation, thus obtaining a “voltage-force” relationship during the calibration that reflects the forces produced by the tibialis anterior during the experiments.


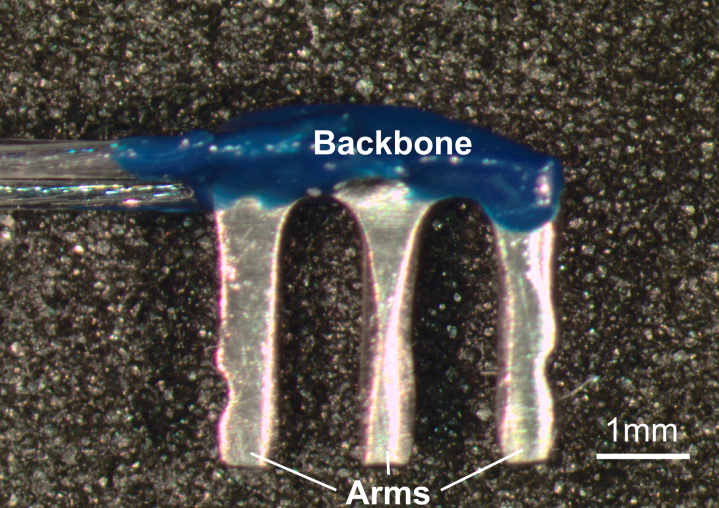


Fig. R20. The miniature E-shaped buckle-type force transducer used in the current study. The transducer consists of a backbone and three arms.


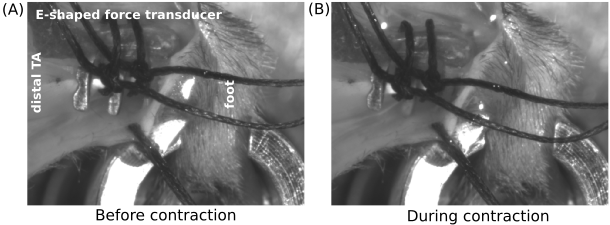


Fig. R21. The movement of the force transducer during a near-maximal muscle contraction. Although the arms of the E-shaped transducer sit on the tibia, the backbone of the transducer was unrestricted and was free to rotate during muscle contraction.
